# Perinatal mortality after the Fukushima nuclear accident: An ecological study

**DOI:** 10.1371/journal.pone.0264491

**Published:** 2022-02-28

**Authors:** Alfred Körblein

**Affiliations:** Untere Söldnersgasse 8, Nürnberg, Germany; University of South Carolina, UNITED STATES

## Abstract

**Objective:**

This study continues former studies on perinatal mortality in Japan after the Fukushima Daiichi nuclear power plant (FDNPP) accident in March 2011. An increased study region is chosen, and the study period is extended to 2019.

**Methods:**

Japanese monthly perinatal mortality data are provided on a prefecture level by the Japanese government. The study region consists of 12 prefectures around the FDNPP; the rest of Japan is used as the control region. A combined non-linear regression of perinatal mortality rates in the study- and control regions is conducted. The regression model allows for a common asymptotic lower limit of perinatal mortality, seasonal variations, and periodic peaks in 2012–2019 in the study region. To determine the dependency of the effect on distance from the FDNPP, the study region is divided into four core prefectures and eight prefectures surrounding the core prefectures.

**Results:**

Perinatal mortality rates in the study region show a significant 6.4% (95% CI: 1.8%, 13.4%) overall increase in 2012–2019 relative to the trend in preceding years with no attenuation during 2012–19. The increase translates to 590 (165, 1226) excess perinatal deaths (p = 0.016). It is characterized by annual peaks with maxima in April. A 13.6% increase is determined in the four core prefectures and a 4.3% increase in eight prefectures surrounding the core prefectures. Before 2012, there is a peak around April 2011 and a decline in October 2011; another significant peak is detected in November 2012. In the 4 core prefectures, large increases are found in the first quarter of 2018 (+70%) and in May 2019 (+130%).

**Conclusion:**

This study finds periodic peaks in perinatal mortality in spring 2012–2019 in 12 prefectures of Japan surrounding the FDNPP. In light of massive increases in 2018 and 2019 in the four core prefectures, continued investigation of perinatal mortality in contaminated regions of Japan is recommended.

## Introduction

After the accident at the Fukushima Daiichi nuclear power plant (FDNPP) in March 2011, little attention was paid to possible adverse effects on pregnancy outcomes in Fukushima and neighboring prefectures. The 2013 UNSCEAR report on Fukushima stated that prenatal exposures from the accident at FDNPP “were not expected to increase the incidence of spontaneous abortions, miscarriages, perinatal mortality, congenital effects or cognitive impairment” [1], although there were several reports on teratogenic radiation effects after the Chernobyl accident in 1986. An official German study [2], conducted by the Federal Office of Radiation Protection (BfS), investigated perinatal mortality rates in Bavaria which was the German state with the highest mean cesium soil contamination. In the southern part of Bavaria (south of River Danube), the cesium soil contamination was three times higher than north of River Danube. Any increase in perinatal mortality after Chernobyl relative to the expected trend should have been greater in southern Bavaria than in northern Bavaria. In both parts of Bavaria, there was no significant excess in perinatal mortality in any of eight 3-month periods from June 1986 through May 1988. However, a trend analysis of German annual perinatal mortality rates found a statistically significant 5% increase in 1987, one year after the Chernobyl disaster [3]. The result was later confirmed by Scherb et al. [4]. The analysis of the monthly data detected peaks in perinatal mortality rates at the beginning and end of 1987 which were associated with peaks of cesium concentration in pregnant women, lagged by seven months [3]. The effects were interpreted as teratogenic radiation effects.

After the Fukushima nuclear accident in March 2011, a survey of stillbirth rates, pre-term births, low birth weight, and congenital anomalies by the Fukushima Radiation Medical Science Center did not find significant regional differences in stillbirth rates within Fukushima Prefecture [5]. Scherb et al. examined the trend of perinatal mortality before and after the Fukushima nuclear accident in six prefectures near the Fukushima Daiichi nuclear power plant (FDNPP) [6]. Using linear logistic regression, they determined a highly significant upward shift in perinatal mortality rates in January 2012 through December 2014 relative to the expected trend before 2012. Körblein and Küchenhoff [7] conducted a combined regression of annual perinatal mortality rates in a study region (Fukushima Prefecture plus 6 nearby prefectures) and the rest of Japan (the control region). To model the long-term trend of perinatal mortality, they used individual exponential trends with a common constant that represented a natural lower limit of perinatal mortality. They found a significant upward shift in perinatal mortality in 2012–2015, but the effect was substantially smaller than reported in [6]. A subsequent study by the same authors used monthly data of perinatal mortality through December 2017 from a study region consisting of Fukushima plus four adjacent prefectures; the rest of Japan served as the control region [8]. In 2012–2017, periodic peaks in perinatal mortality were detected with maxima in April. In Fukushima Prefecture, the effect was 3-times greater than in the four neighboring prefectures.

In the present study, the same statistical methods as in [8] are applied, but with data up to 2019 and with an expanded study region (12 prefectures). To determine the dependency of the effect size on distance from the FDNPP, the study region is subdivided into four prefectures near the FDNPP (Area A) and eight surrounding prefectures (area B). The enlarged study region allows a more accurate estimate of the overall impact of the Fukushima accident on perinatal mortality in Japan. These 12 prefectures were used in a recent study by the present author reporting a highly significant decrease in live births in December 2011, nine months after the Fukushima accident [9].

## Material and methods

The numbers of live births, stillbirths, and early neonatal deaths (first 7 days), 2002 through 2019, are provided on a website of the Japanese government [10]. Stillbirths are defined in Japan as fetal deaths after 22 weeks of pregnancy. Perinatal mortality is defined as the number of stillbirths plus early neonatal deaths, divided by the number of live births plus stillbirths.

**Fig 1:**
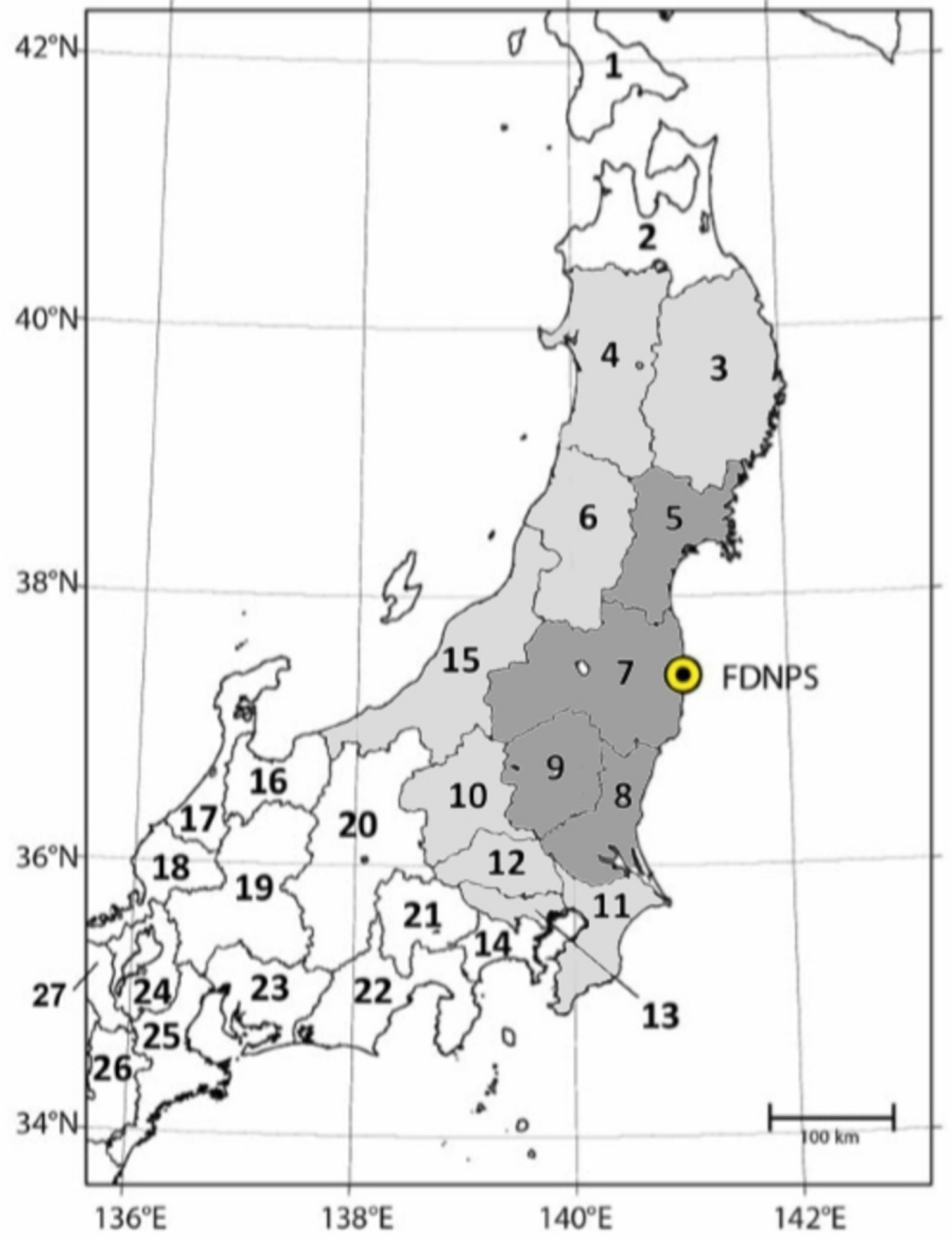
Map of prefectures around Fukushima Daiichi nuclear power station (FDNPS). Area A (shaded dark grey): Prefectures Fukushima (7), Miyagi (5), Tochigi (9), Ibaraki (8); area B (shaded light grey): Prefectures Iwate (3), Akita (4), Yamagata (6), Niigata (15), Gunma (10), Saitama (12), Tokyo (13), Chiba (11). From UNSCEAR 2013 [1], Annex A, Figure VI.

The prefectures of Fukushima plus three adjacent prefectures (Miyagi, Ibaraki, and Tochigi) are chosen as the core area (Area A). The surrounding eight prefectures (Iwate, Akita, Yamagata, Niigata, Gunma, Saitama, Tokyo, and Chiba) are used for comparison (area B). The remaining 35 prefectures of Japan constitute the control region (area C). A map of the study region is provided in Fig 1. The division of the study region is based on distance from the FDNPP rather than on radiation exposure.

Table 1 contains the numbers of live births (LB), stillbirths (SB), and early neonatal deaths (NEO) in areas A, B, and C for two periods, 2002–2011 and 2012–2019.

**Table 1:** Numbers of live births, stillbirths, and early neonatal deaths by area and period.

	Area A	Area B	Area C
2002–2011			
LB	794,052	2,789,382	7,333,071
SB	3016	10,898	26,511
NEO	828	2600	7171
rate per 1000	4.82	4.82	4.58
2012–2019			
LB	531,483	2,078,425	5,173,037
SB	1703	6223	14,914
NEO	425	1399	3587
rate per 1000	3.99	3.66	3.57

### Statistical analysis

First, a combined analysis of the data from the study region (12 prefectures) before March 2011 and the data from the rest of Japan (control region) in 2002–2019 is conducted. To model the time trends in the study- and control regions, exponentials with a constant of the form *y* = *α*+*exp*(*β*_1_+*β*_2_·*t*) are used, where *t* is time, *β*_1_ and *β*_2_ are individual trend parameters, and constant *α* is a natural lower limit of perinatal mortality which is assumed to be equal in the study- and control region. Under the Null-hypothesis of no effect of the Fukushima accident on perinatal mortality in the study region, Model (1) has the following form:

$$E(y(t))=\alpha +\exp\left(\beta_{1}+\beta_{2}∙study+\beta_{3}∙t+\beta_{4}∙t\cdot study\right)$$ (1.0)

Here, *E*(*y*(*t*)) is the expected value of perinatal mortality *y*(*t*); time *t* is defined as calendar year minus 2000, in fractions of a year (e.g. mid-January 2002 is *t* = 2+1/24), and dummy variable *study* denotes the data from the study region. The number of excess perinatal deaths in March 2011 through December 2019 is determined from the difference between observed and predicted deaths. This analysis is free of assumptions about trends in perinatal mortality after the Fukushima accident.

Next, a regression allowing for a level shift in January 2012 is conducted. The regression model is adopted from [8]. An indicator variable *cp* (change point) is defined with *cp* = 1 in January 2012 through December 2019 and *cp* = 0 before January 2012 and in the control region. Model (1.1) has the following form:

$$E(y(t))=\alpha +\exp\left(\beta_{1}+\beta_{2}∙study+\beta_{3}∙t+\beta_{4}∙t\cdot study+cp∙(\beta_{5}+\beta_{6}∙(t-12))\right)$$ (1.1)

Parameter *β*_5_ estimates the size of the level shift in January 2012, and *β*_6_·(*t*−12) allows for a slope change in January 2012, i.e. an attenuation of the effect during 2012–2019.

Third, the regression model controls for seasonal variations and allows for periodic peaks of perinatal mortality in 2012–2019. Seasonality, as well as periodicity, is modeled by pairs of sine and cosine terms with periods of 6 and 12 months. Model (1.2) has the following form:

$$E(y(t))=(\alpha +\exp(\beta_{1}+\beta_{2}∙study+\beta_{3}∙t+\beta_{4}∙t\cdot study+cp∙((\beta_{5}+\beta_{6}∙(t-12))∙(1+\beta_{7}∙\sin(2\pi ∙t)+\beta_{8}∙\cos(2\pi ∙t)+\beta_{9}∙\sin(4\pi ∙t)+\beta_{10}∙\cos(4\pi ∙t))))\cdot (1+{\beta_{11}\cdot \sin\left(2\pi \cdot t\right)+\beta }_{12}∙\cos(2\pi ∙t)+\beta_{13}∙\sin(4\pi ∙t)+\beta_{14}∙\cos(4\pi ∙t))$$ (1.2)

### Exploratory analysis

The moving average of the residuals after the Fukushima accident exhibits noticeable peaks around April 2011 and November 2012, as well as a trough in October 2011. To avoid distortion of the long-term trend of the data, Model (1.2) is complemented by three bell-shaped excess terms (normal distributions) to fit the two peaks and the trough. Model (1.3) has the following form:

E(y(t))=(α+exp(β1+β2·study+β3·t+β4·t·study+cp∙((β5+β6·(t−12))∙(1+β7∙sin(2π∙t)+β8∙cos(2π∙t)+β9∙sin(4π∙t)+β10∙cos(4π∙t))+β11/exp((t−β12)^2/2/β13^2))))·(1+β14∙sin(2π∙t)+β15∙cos(2π∙t)+β16∙sin(4π∙t)+β17∙cos(4π∙t))+study·(β18/exp((t−β19)^2/2/β20^2)+β21/exp((t−β22)^2/2/β23^2))) (1.3)

To test the significance of the periodic terms in 2012–2019, the deviance obtained with Model (1.3) is compared with the deviance resulting from a regression without the periodic peaks. The p-value is determined by an F-test.

### Combined regression of data from areas A, B, and C

To test whether there is a difference in effect size between areas A and B over the 2012–2019 period, data from Areas A, B, and C are analyzed in a combined regression. A change-point model analogous to Model (1.1) is used, with individual trend parameters (intercepts and slopes) in areas A, B, and C, and individual level shifts (*cp*) in areas A and B, but with a common slope change in January 2012 in areas A and B. Model (2.1) has the following form:

$$E(y(t))=\alpha +\exp\left(\beta_{1}+\beta_{2}∙A+{\beta_{3}∙B+\beta }_{4}∙t+\beta_{5}∙t\cdot A+\beta_{6}∙t\cdot B+cp∙{(\beta }_{7}∙A+\beta_{8}∙B\right)+\beta_{9}\cdot (t-12)))$$ (2.1)

Next, Model (2.2) is applied which allows for seasonal variations and periodic peaks in 2012–2019 in areas A and B, analogous to Model (1.2):

$$E(y(t))=(\alpha +\exp(\beta_{1}+\beta_{2}∙A+\beta_{3}∙B+\beta_{4}∙t+\beta_{5}∙t\cdot A+\beta_{6}∙t\cdot B+cp∙({(\beta }_{7}\cdot A+\beta_{8}∙B+\beta_{9}\cdot (t-12))∙(1+\beta_{10}∙\sin(2\pi ∙t)+\beta_{11}∙\cos(2\pi ∙t)+\beta_{12}∙\sin(4\pi ∙t)+\beta_{13}∙\cos(4\pi ∙t)))))\cdot (1+{\beta_{14}\cdot \sin\left(2\pi \cdot t\right)+\beta }_{15}∙\cos(2\pi ∙t)+\beta_{16}∙\sin(4\pi ∙t)+\beta_{17}∙\cos(4\pi ∙t))$$ (2.2)

### Exploratory analysis

The trend of perinatal mortality in Area A exhibits striking peaks in the first quarter of 2018 and in May 2019. To avoid distortion of the long-term trend, a dummy variable *cp*18 is added to Model (2.2). It is defined as cp18 = 1 in Jan 2018 through Dec 2019 in Area A and cp18 = 0 otherwise. In addition, bell-shaped excess terms are added to allow for the effects in April 2011, October 2011, and November 2012. A common parameter is used for the width of the bell-shaped excess terms since the regression with Model (1.3) had shown that they agreed within the limits of error. Model (2.3) has the following form:

E(y(t))=(α+exp(β1+β2·A+β3·B+β4·t+β5·t·A+β6·t·B+cp12∙((β7·A+β8·B)·(1+β9∙sin(2π∙t)+β10∙cos(2π∙t)+β11∙sin(4π∙t)+β12∙cos(4π∙t))+β13·(t−12)+β14·A·cp18+(β15·A+β16·B)/exp((t−β17)^2/2/β18^2))))·(1+β19∙sin(2π∙t)+β20∙cos(2π∙t)+β21∙sin(4π∙t)+β22∙cos(4π∙t)+(β23·A+β24·B)/exp((t−β25)^2/2/β18^2)+(β26·A+β27·B)/exp((t−β28)^2/2/β18^2)) (2.3)

Iteratively reweighted non-linear regression with program R, function *nls*(), is used for data analysis and plotting, and a two-sided p-value <0.05 is considered statistically significant.

## Results

### Study region

The results of the combined regression of the data from the study- and control regions with Model (1.0), i.e. parameter estimates, standard errors, t-values, and p-values, are listed in S1 Table in S1 File. S1 Fig in S1 File shows the perinatal mortality rates in the study- and control region and the regression lines. With O = 10,931 observed and E = 10,225 predicted perinatal deaths, 706 excess perinatal deaths are identified from March 2011 to December 2019, representing an overall increase of 6.9%. For 2012–2019, the corresponding figures are O = 9750 and E = 9079 which means 671 excess perinatal deaths and a 7.4% increase.

**Fig 2:**
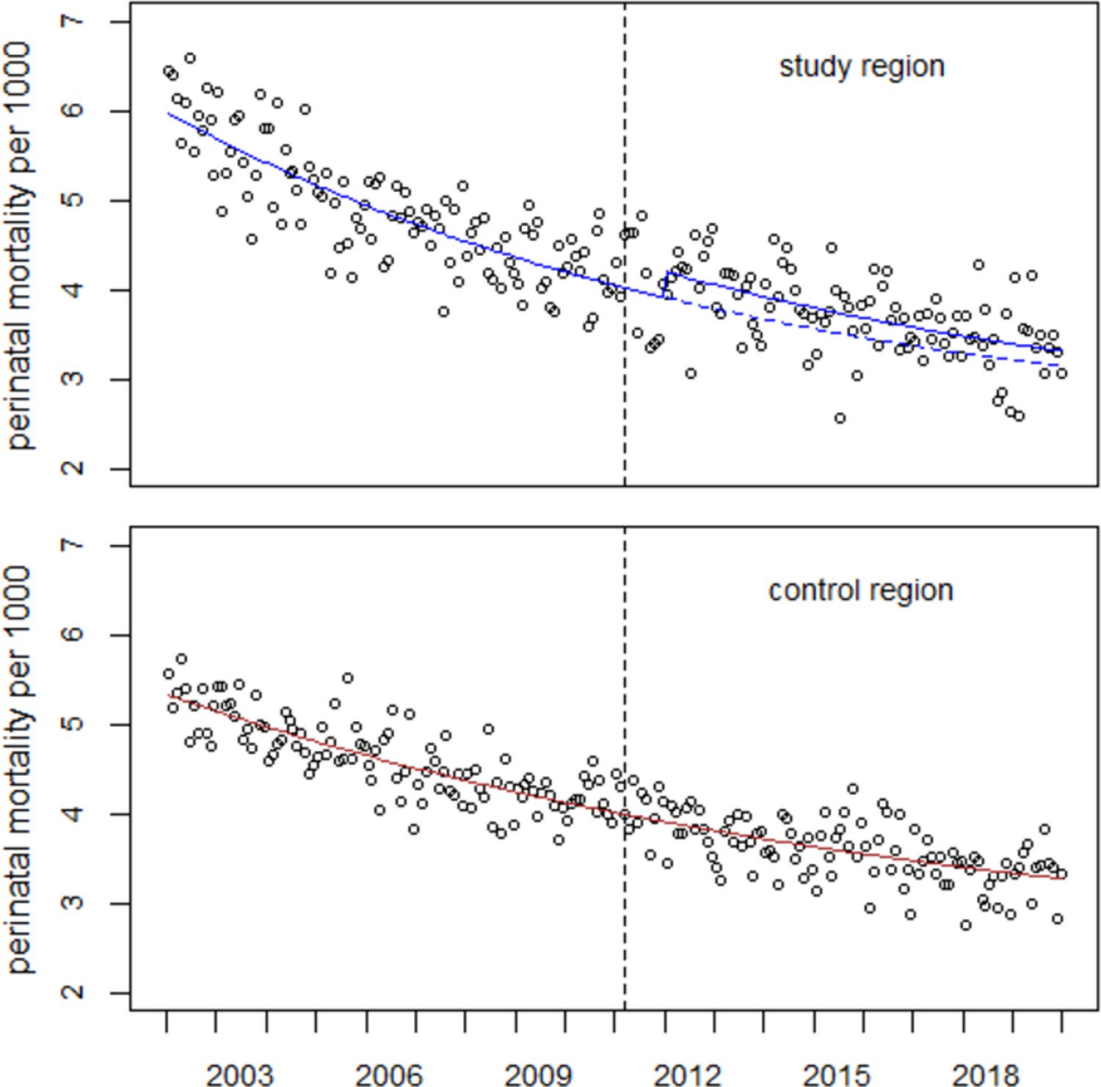
Perinatal mortality in the study- and control regions and result of combined regression with Model (1.1). The broken line shows the undisturbed trend in 2012–2019.

The regression with Model (1.1)—which includes a level shift (cp) and a slope change in January 2012 in the study region—leads to a significant improvement in the fit compared to a regression without level shift and slope change (p = 0.016, F-test). The estimated number of excess perinatal deaths from 2012–2019 is 590 (95% CI: 165, 1226), an overall increase of 6.4% (1.8%, 13.4%). The regression results are presented in Table 2 and Fig 2.

**Table 2:** Results of regression of data from the study region with Model (1.1).

parameter	estimate	SE	t-value	p-value
α	0.0023	0.0004	6.186	<0.001
β1	-5.6778	0.0866	-65.60	<0.001
β2	0.2321	0.0435	5.340	<0.001
β3	-0.0641	0.0138	-4.655	<0.001
β4	-0.0194	0.0060	-3.239	0.001
β5	0.1671	0.0692	2.415	0.016
β6	0.0028	0.0167	0.165	0.869

y(t)~ α+exp (β1+β2·study+β3·t+β4·t·study+ β5·cp+β6·(t-12))

Deviance = 464.83 (df = 425).

Regression with Model (1.2), which controls for seasonal variations in 2002–2019 and allows for periodic annual peaks in the study region in 2012–2019, reduces the deviance from 464.8 (df = 425) to 442.1 (df = 417), p = 0.007. The regression results are presented in S2 Table and S2 Fig in S1 File.

Regression with Model (1.3) reduces the deviance from 442.1 (df = 417) to 422.6 (df = 408), p = 0.029 (F-test).

To test the significance of the periodic terms in 2012–2019, the deviance obtained with Model (1.3) is compared with the deviance resulting from a regression without the periodic peaks. The deviance is 431.8 (df = 412) without and 422.6 (df = 408) with the periodic terms (p = 0.065, F-test).

Table 3 shows the improvement of the fit with stepwise model refinement. The overdispersion factor (OD), i.e. the deviance divided by the degrees of freedom (df), is used as a criterion of the goodness of fit. OD decreases with each step and reaches a value of 1.036 with Model (1.3). In Table 3, P is the number of parameters added to the model with each step of model refinement, and the p-values are the results of F-tests with (P, df) degrees of freedom.

**Table 3:** Improvement of fit to the data from the study region by stepwise model refinement.

step	Description	df	deviance	OD^a^	P^b^	F-value	p-value
0	Null-hypothesis	427	474.0	1.110			
1	Level shift in 2012	425	464.8	1.094	2	4.184	0.016
2	Seasonal effect	421	447.7	1.063	4	4.039	0.003
3	Periodic peaks	417	442.1	1.060	4	1.314	0.264
4	Bell-shaped terms	408	422.6	1.036	9	2.093	0.029

^a^ OD = Overdispersion = deviance / df

^b^ P = Number of parameters added with each step.

**Fig 3:**
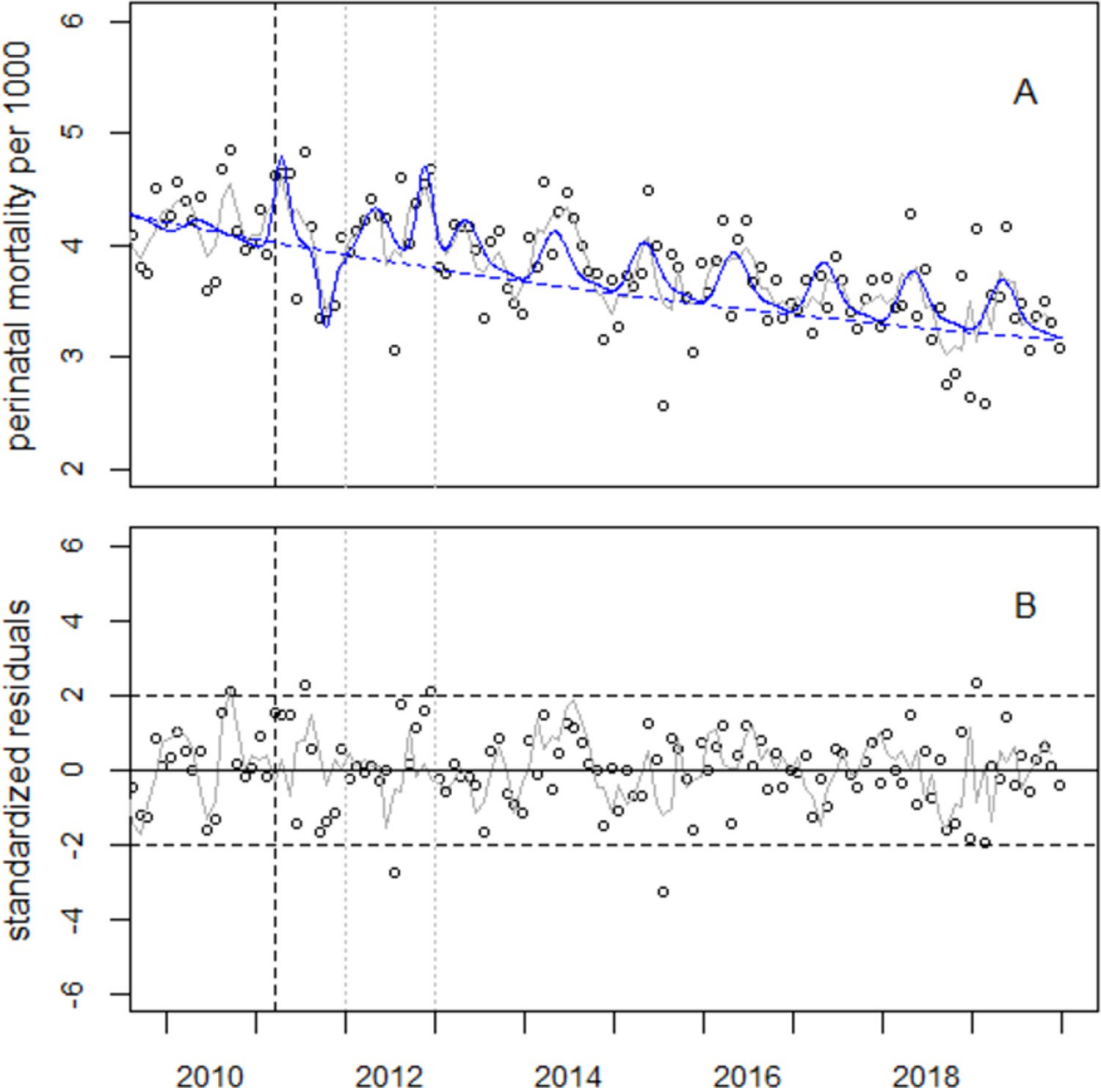
Panel A: Perinatal mortality in the study region and result of regression with Model (1.3). Panel B: Residuals in units of standard deviations (standardized residuals). The grey lines show the 3-month moving averages.

With 9750 observed (O) and 9167 expected (E) perinatal deaths in 2012–2019, Model (3.1) yields 583 (158, 1016) excess perinatal deaths in 2012–2019 which translates to an overall increase of 6.4% (1.7%, 11.1%). The regression results with Model (1.3) are listed in S3 Table in S1 File and shown in Fig 3.

The estimate of parameter *α* (the lower limit of perinatal mortality) is 2.35 ± 0.35 per 1000 (p<0.001). The points in time of the bell-shaped excess terms are found in April 2011 (β_19_ = 11.279 ± 0.047), October 2011 (β_22_ = 11.776 ± 0.045), and November 2012 (β_12_ = 12.890 ± 0.043). The effect sizes are β_18_ = 0.180 ± 0.100 (p = 0.075) for the peak in April 2011, β_21_ = -0.168 ± 0.086 (p = 0.050) for the trough in October 2011, and β_11_ = 0.456 ± 0.196 (p = 0.020) for the peak in November 2012. The standard deviations of the bell-shaped terms agree within the limits of error (β_20_ = 0.075 ± 0.047, β_23_ = 0.072 ± 0.045, and β_13_ = 0.098 ± 0.043, respectively).

Eventually, the standardized residuals (see Fig 3 panel B) are checked for autocorrelation. There is no noticeable autocorrelation in the data from the study region nor from the control region for time lags of 1 to 11 months (see S3 Fig in S1 File).

### Combined regression

**Fig 4:**
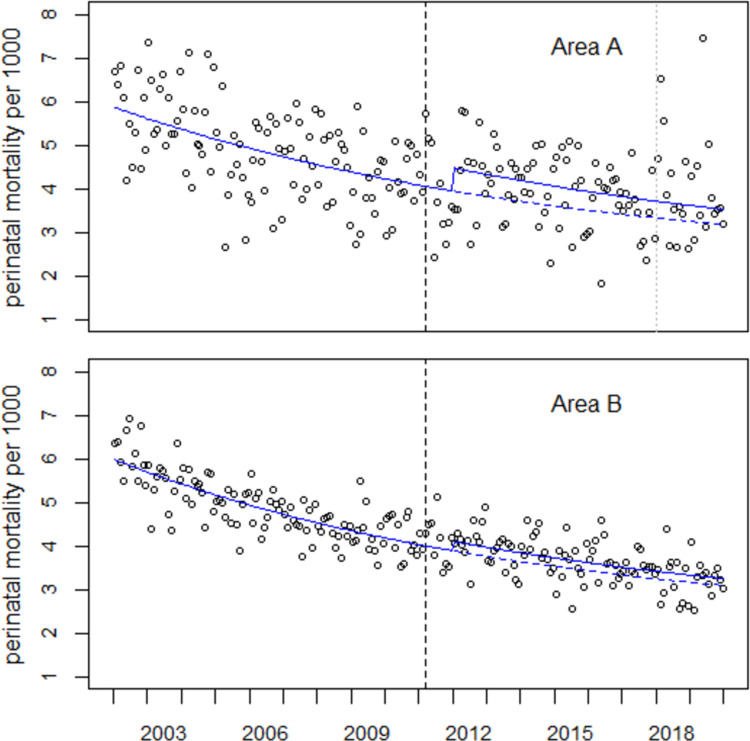
Perinatal mortality rates in areas A and B and result of a combined regression with Model (2.1) (solid lines). The broken lines show the predicted undisturbed trends.

The results of the combined regression of the data from areas A, B, and C with Model (2.1), which allows for individual level shifts in areas A and B in January 2012, are listed in Table 4 and shown in Fig 4. From the difference between observed (O = 2128) and predicted (E = 1891) perinatal deaths, 237 excess perinatal deaths are determined in 2012–2019 in Area A, a relative increase of 12.5%. The respective numbers for area B are O = 7622, E = 7232, O-E = 390, (O-E)/E = 0.054. The ratio of the increases in areas A and B is 0.125/0.054 = 2.32. Altogether, 627 excess perinatal deaths are determined in 2012–2019.

**Table 4:** Results of combined regression of data from areas A, B, C with Model (2.1).

parameter	estimate	SE	t-value	p-value
α	0.0022	0.0004	5.458	<0.001
β1	-5.6584	0.0948	-59.671	<0.001
β2	0.1919	0.0735	2.613	0.009
β3	0.2393	0.0475	5.035	<0.001
β4	-0.0611	0.0137	-4.472	<0.001
β5	-0.0142	0.0109	-1.297	0.195
β6	-0.0209	0.0063	-3.317	<0.001
β7	0.2683	0.1281	2.095	0.037
β8	0.1243	0.0703	1.768	0.078
β9	0.0058	0.0154	0.379	0.705

y(t) ~ α+exp (β1+β2·A+β3·B+β4·t+β5·t·A+β6·t·B+cp12·(β7·A+β8·B+β9·(t-12)))

Deviance = 710.87 (df = 638).

A regression with Model (2.2), which allows for seasonal effects and periodic peaks in 2012–2019, yields a significant (p = 0.002) improvement of the fit over Model (2.1). Seasonal effects reduce the deviance from 710.9 to 693.1 (p = 0.003), and the periodic peaks in 2012–2019 result in a deviance of 684.2 (p = 0.084). The regression results are provided in S4 Table and S4 Fig in S1 File.

### Exploratory analysis

The regression results with Model (2.3) are listed in S5 Table in S1 File. Table 5 shows the improvement of the model fit by stepwise model refinement. The overdispersion factor (OD) decreases with each step. Step 0 refers to the model without a level change in January 2012. A major improvement of the fit results from the level shift in Area A in 2018–2019 (step 4, p = 0.004). The bell-shaped excess terms reduce the deviance from 675.0 (df = 629) to 652.2 (df = 619), p = 0.018. Regression with Model (2.3) yields 596 excess perinatal deaths in 2012–2019. In comparison, the simple change point model Model (1.1) identified 590 additional cases.

**Table 5:** Improvement of fit with stepwise model refinement.

Step	Description	df	deviance	OD^a^	P^b^	F-value	p-value
0	Null hypothesis	641	722.1	1.126			
1	Level change in 2012	638	710.9	1.114	3	3.35	0.019
2	Seasonal effects	634	693.1	1.093	4	4.07	0.003
3	Periodic peaks	630	683.8	1.085	4	2.15	0.073
4	Level change in 2018	629	675.1	1.073	1	8.05	0.005
5	Bell-shaped terms	619	652.2	1.054	10	2.17	0.018

^a^ OD = Overdispersion = deviance / df

^b^ P = Number of parameters added with each step.

An inspection of the list of parameters in S5 Table in S1 File shows that both the intercepts (parameters β_2_ and β_3_) and the slopes (parameters β_5_ and β_6_) in Areas A and B agree within the limits of error; the differences are negligible (p = 0.93 and p = 0.79, respectively). Moreover, there is no notable attenuation of the effect in 2012–1019; the estimate of the slope change is β_2_ = 0.002 ± 0.013 (p = 0.89). Therefore, Model (2.3) is replaced by a more parsimonious and robust regression Model (2.4) with common parameters for the intercepts and slopes in areas A and B and without a slope change in January 2012. Regression with Model (2.4) yields slightly lower deviance (652.0) than model (2.3), although it requires three fewer parameters. The overdispersion factor (OD) decreases from 1.054 to 1.048 so the variance is only 5% larger than in a purely random distribution.

The results of the regression with Model (2.4) are listed in S6 Table in S1 File. The estimate of the natural lower limit of perinatal mortality (parameter α) is 2.35 ± 0.28 per 1000 (p<0.001). The level change in January 2012 is estimated as β_7_ = 0.251 ± 0.085 (p = 0.003) in Area A and β_8_ = 0.100 ± 0.053 (p = 0.057) in area B. The difference in effect size is statistically significant (p = 0.020). The effect of the peak in April 2011 is significant in Area A (β_23_ = 0.42 ± 0.21, p = 0.047), but not in area B (β_24_ = 0.13 ± 0.09, p = 0.22). This is also the case for the trough in October 2011 (β_26_ = -0.32 ± 0.15, p = 0.032 and β_27_ = -0.13 ± 0.09, p = 0.13, respectively). In contrast, the peak in November 2012 is smaller in Area A (β_15_ = 0.30 ± 0.39; p = 0.44) than in area B (β_16_ = 0.54 ± 0.21; p = 0.010).

To check the statistical significance of periodicity in 2012–2019, the deviance obtained with Model (2.4) is compared with the deviance resulting from a regression without the periodic terms. With deviance = 652.0 (df = 622) and deviance = 663.1 (df = 626) respectively, the effect of periodicity in 2012–2019 is statistically significant (p = 0.032, F-test).

Table 6 shows the numbers of excess deaths in areas A and B before and after 2018. In 2012–2017, the ratio of the increases in areas A and B is 2.11 (10.9% vs. 5.2%) whereas, in 2018–2019, there is a large 24% increase in Area A, but a negligible 1% increase in Area B.

**Table 6:** Excess perinatal deaths in areas A and B in 2012–2017 and 2018–2019.

2012–2019	O	E	O-E	O/E	RR^a^
Area A	2128	1873.1	254.9	1.136	
Area B	7622	7310.4	311.6	1.043	
sum	9750	9183.5	566.5	1.062	3.19
2012–2017					
Area A	1650	1488.5	161.5	1.109	
Area B	6041	5745.0	296.0	1.052	
sum	7691	7210.3	457.5	1.063	2.11
2018–2019					
Area A	478	384.6	93.4	1.243	
Area B	1581	1565.4	15.6	1.010	
sum	2059	1950.0	109.0	1.056	

^a^RR: Relative risk (O-E)/E in Area A divided by (O-E)/E in Area B.

**Fig 5:**
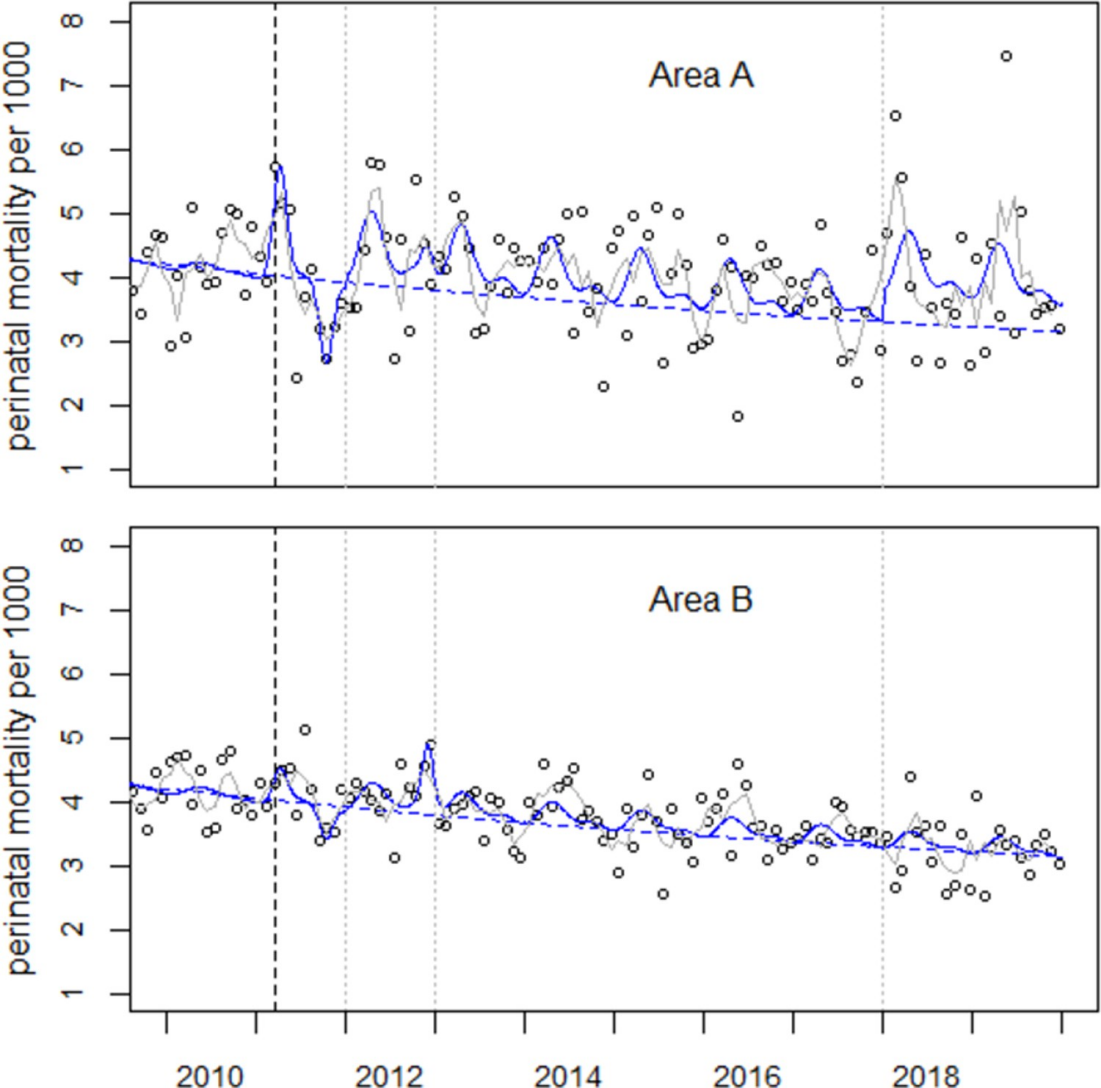
Perinatal mortality in areas A and B and regression results with Model (2.3).

The estimated total number of excess perinatal deaths in 2012–2019 with Model (2.3) is 567, somewhat less than resulting from a regression with Model (2.2) with 596 excess cases. Fig 5 shows the trends of perinatal mortality rates in areas A and B and the respective regression lines with Model (2.3). Most striking are the peaks in Area A in the first quarter of 2018 (O = 83, E = 48.6, O/E = 1.71) and in May 2019 (O = 38, E = 16.1, O/E = 2.36). In Area B, the greatest increase is observed in November 2012.

As with the data from the study- and control region, the standardized residuals are checked for autocorrelation. For time lags of 1 to 11 months, no significant autocorrelation can be seen either in Area A or in Area B (see S5 Fig in S1 File).

### Sensitivity analyses

Below, the extent is examined to which the peak in April 2011 and trough in October 2011 affect the number of excess perinatal deaths in 2012–2019. First, a regression is carried out without adjusting for the effect in April 2011. The regression yields 474 excess cases in 2012–2019, substantially less than the 583 excess cases obtained with the full Model (1.3). In turn, a regression without the trough in October 2011 yields 728 excess deaths in 2012–2019.

Eventually, the method used by Scherb et al. in [6] is applied to the present data from the study region. Linear logistic regression was used in [2] with a level shift in January 2012 and a dummy variable for March-May 2011. With this model, the estimated number of excess perinatal deaths in 2012–2019 is 996 (530, 1641), see S6 Fig in S1 File. Regression with a linear-quadratic trend fits the data better than a linear trend (p = 0.002), and the estimated number of excess perinatal deaths decreases to 752. This is not much different from the 728 excess cases identified above with the regression neglecting the October 2011 trough.

## Discussion

The present study uses a larger study region (12 prefectures) and a longer study period (2012–2019) than previous studies of perinatal mortality after the accident at the Fukushima Daiichi nuclear power plant (FDNPP). It finds an overall 6.4% increase in perinatal mortality in the study region in 2012–2019 with 590 excess perinatal deaths. The increase is 3.2-times greater in four core prefectures than in eight surrounding prefectures. The effect in 2012–2019 is characterized by periodic peaks with maxima in April. Significant excess mortality is found in 2018 and 2019 in Area A, driven by large peaks in the first quarter of 2018 (+70%) and in May 2019 (+130%).

Regular peaks in perinatal mortality in April were also reported in [8] which analyzed data through 2017. There, the authors suggested a possible link between the peaks in perinatal mortality and mushroom consumption in the preceding autumn (see S7 Fig in S1 File). Unpublished research of the Chernobyl consequences by the present author found peaks in April 1987 in perinatal mortality data from the Ukrainian oblast Zhytomyr (see S8 Fig in S1 File) and in infant mortality data from Poland (see S9 Fig in S1 File). In Bavaria, the German region with the highest fallout from Chernobyl, perinatal mortality peaked in May 1987 (see S10 Fig in S1 File). The corresponding data sets are provided in S7 Table through S9 Table in S1 File.

In addition to periodic peaks in April 2012–2019, a peak in perinatal mortality was observed in April 2011, followed by a trough in October 2011. Another significant increase was detected in November 2011. Fitting these effects by bell-shaped excess terms was intended to eliminate distortion of the long-term trend of the data. Possible explanations for the observed effects are suggested below.

1. The peak in April 2011 might be an immediate effect of the earthquake and tsunami. To check this hypothesis, regressions of perinatal mortality rates in the four prefectures of Area A are conducted with a dummy variable for March 2011. The increases in March 2011 are greater in the three coastal prefectures (Miyagi: +85%, Fukushima: 84%, and Ibaraki: +33%) than in the inland prefecture Tochigi (+20%, p = 0.61). The trends of the data in the four prefectures are presented in S11 and S12 Figs in S1 File.
2. The observation of a drop in perinatal mortality rates in October 2011 is unexpected; one would rather expect to see an increase in perinatal mortality seven months after the Fukushima accident. However, the radiation burst in March 2011 might have caused selective spontaneous abortions of the most radiosensitive embryos, resulting in a more radioresistant surviving fraction. Interestingly, the ratio of the effects in October 2011 in Area A (β_26_ = -0.321) and Area B (β_27_ = -0.128) is the same (2.5) as the ratio of the level shifts in Area A (β_7_ = 0.251) and Area B (β_8_ = 0.100), suggesting a common cause (e.g. radiation exposure) for these effects. In Japan, spontaneous abortions are only registered after 14 weeks of pregnancy, so the only way to find out about earlier abortions is to look at the trend of live births. In fact, Japanese monthly data of LB show a significant 2.7% drop in October 2011 (p = 0.006). The birth deficit is greater for males (-3.9%, p = 0.003) than for females (-1.5%, p = 0.25) which leads to a significant (p = 0.008) drop in the sex ratio at birth (male/female births) in Oct 2011, see S13-S15 Figs in S1 File. A similar drop in sex ratio was found in Czechia in November 1986, 7 months after the Chernobyl accident [11]. Greater radio-sensitivity of male than female embryos could explain the distorted sex ratio.
3. The peak in the study region in November 2012 is consistent with a peak in perinatal mortality in November 1987 in Germany after the Chernobyl accident (see S16 Fig and S10 Table in S1 File). The November 1987 peak was associated with an increase in cesium burden in pregnant females during the winter of 1986/87 when cows were fed contaminated silage from the summer of 1986 [3]. In Germany, the main path for cesium was the consumption of cow milk and dairy products. In Japan, the average per capita consumption of cow milk is substantially lower than in Germany (36 vs. 91 kg per year in 2002 [12]), but milk consumption may be greater in urban regions than in rural areas. This could explain why the November 2012 peak is smaller in Area A than in Area B which includes Tokyo.

### Strengths and limitations

This study uses a larger study area (12 prefectures) and a longer study period (2002–2019) than previous studies of perinatal mortality after Fukushima [5–8]. In [8], the effect size in Fukushima prefecture was compared with that in four adjacent prefectures whereas the present study uses four core prefectures and eight surrounding prefectures for comparison. Due to much larger case numbers, any dependency of the effect on distance (as a proxy of dose) from the FDNPP can be determined more precisely. The remaining 35 Japanese prefectures are used as a control region. Thus, the study includes all perinatal deaths in Japan during 18 years; it has the greatest possible statistical power. An initial confirmatory analysis uses a predefined regression model while the subsequent exploratory analysis controls for observed short-term deviations from the regression model. Possible explanations for these deviations are suggested.

The main limitation of the study is its ecological design; the data are highly aggregated, so causal interpretations of the results are not possible. Interpretation of the observed increase in perinatal mortality as a radiation effect is complicated by the fact that, according to conventional wisdom, teratogenic effects are not expected to occur below a threshold dose of 100 mSv (see e.g. ICRP Publication 90 [13]). According to the UNSCEAR 2013 report [1], Annex A, Table 5, the mean estimated radiation dose to adults in the first year after the Fukushima accident was 1.0–4.3 mSv in Fukushima prefecture and 0.2–1.4 mSv in six neighboring prefectures (Group 3 prefectures).

To conclude, the present study finds a significant increase in perinatal mortality after the Fukushima accident in 12 prefectures of Japan surrounding the FDNPP. The increase is characterized by periodic peaks with maxima in April 2012–2019. In light of the massive increase in 2018 and 2019, continued investigation of perinatal mortality in contaminated regions of Japan is recommended.

## Supporting information

S1 File (PDF)
