# Supplementary material for: Perinatal mortality after the Fukushima nuclear accident: An ecological study
Source: PLoS One. 2022 Feb 28;17(2):e0264491. doi: 10.1371/journal.pone.0264491 (PMC8884545; doi:10.1371/journal.pone.0264491)
Supplement: S1 File — (PDF) [file pone.0264491.s001.pdf]

Supporting Information

S1_Table. Results of regression of data from the study region with Model (1.0)

$y(t) \sim \alpha+\exp(\beta1+\beta2\cdot study+\beta3\cdot t+\beta4\cdot t\cdot study)$

parameter	estimate	SE	t-value	p-value
α	0.0023	<0.0014	5.926	<0.001
β1	-5.6716	0.0893	-63.51	<0.001
β2	0.2401	0.0440	5.455	<0.001
β3	-0.0631	0.0138	-4.586	<0.001
β4	-0.0214	0.0063	-3.385	<0.001

Deviance=338.3 (df=321)

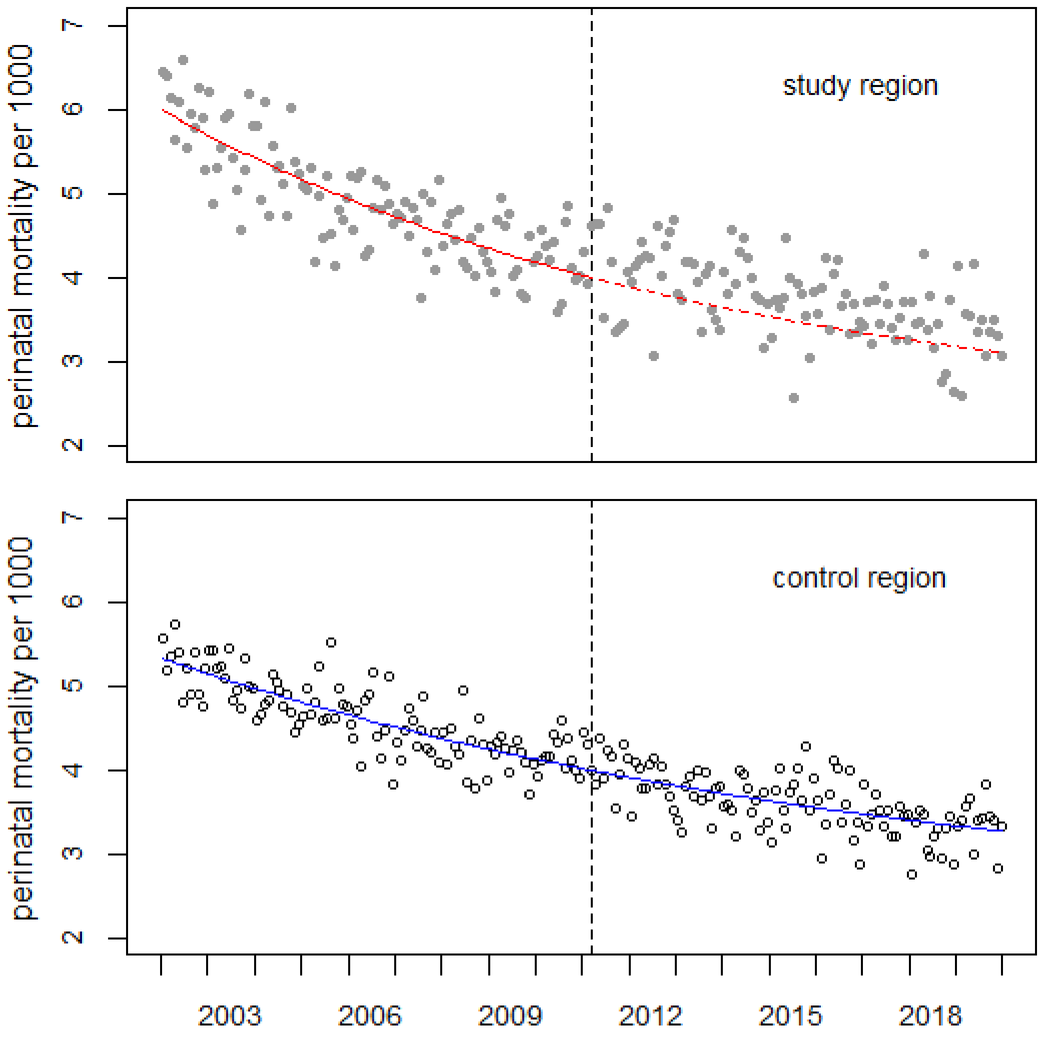

S1_Fig. Perinatal mortality rates in the study- and control region. The solid lines are the results of a combined regression with Model (1.0) of the data from the study region before March 2011 (vertical broken line) and the control region from 2002 to 2019. The broken line is the extrapolated trend in the study region after the Fukushima accident.

S2_Table. Results of regression of data from the study region with Model (1.2)

```
y(t)~ (α+exp (β1+β2·study+β3·t+β4·t·study+cp·((β5+β6·(t-12))
      (1+β7·sin(2π·t)+β8·cos(2π·t)+β9·sin(4π·t)+β10·cos(4π·t)))).
      (1+β11·sin(2π·t)+β12·cos(2π·t)+β13·sin(4π·t)+β14·cos(4π·t))
```

parameter	estimate	SE	t-value	p-value
α	0.0023	<0.0014	5.901	<0.001
β1	-5.6687	0.0894	-63.430	<0.001
β2	0.2296	0.0425	5.404	<0.001
β3	-0.0624	0.0135	-4.639	<0.001
β4	-0.0193	0.0058	-3.345	<0.001
β5	0.1524	0.0655	2.326	0.020
β6	0.0057	0.0155	0.371	0.711
β7	0.0691	0.0442	1.563	0.119
β8	-0.0242	0.0416	-0.581	0.561
β9	-0.0381	0.0420	-0.908	0.365
β10	-0.0304	0.0412	-0.738	0.461
β11	0.0082	0.0055	1.480	0.140
β12	-0.0146	0.0055	-2.648	0.008
β13	-0.0045	0.0055	-0.808	0.420
β14	-0.0023	0.0055	-0.415	0.679

Deviance=442.70 (df=417)

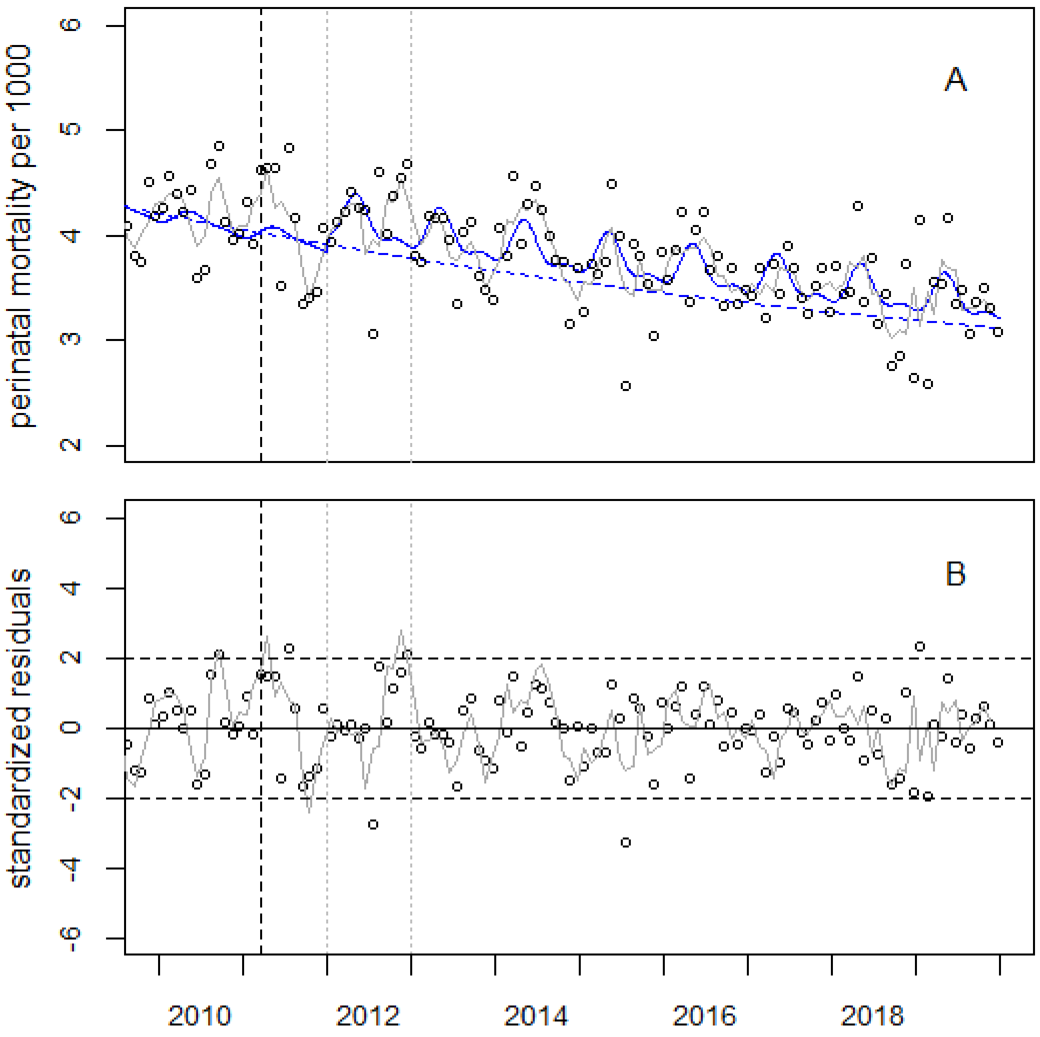

S2_Fig. Panel A: Perinatal mortality rates in the study region and result of regression with Model (1.2). The broken blue line shows the undisturbed trend in 2012-2019. Panel B: Standardized residuals. The grey lines show the 3-month moving averages.

S3_Table. Regression results of data from the study region with Model (1.3)

```
y(t) ~ (α+exp (β1+β2·study+β3·t+β4·tstudy+cp·(β5+β6·(t-12)+
       β7·sin(2π·t)+β8·cos(2π·t)+β9·sin(4π·t)+β10·cos(4π·t)+
       β11/exp(t-β12)^2/2/β13^2)))).
       (1+β14·sin(2π·t)+β15·cos(2π·t)+β16·sin(4π·t)+β17·cos(4π·t)+
       study*(β18·/exp((t-β19)^2/2/β20^2)+β21/exp((t-β22)^2/2/β23^2)))
```

parameter	estimate	SE	t-value	p-value
α	0.0023	<0.0014	6.117	<0.001
β1	-5.6735	0.0869	-65.30	<0.001
β2	0.2309	0.0431	5.354	<0.001
β3	-0.0633	0.0135	-4.702	<0.001
β4	-0.0194	0.0063	-3.095	0.002
β5	0.0897	0.0693	1.293	0.197
β6	0.0176	0.0154	1.142	0.254
β7	0.1084	0.0528	2.053	0.041
β8	-0.0586	0.0473	-1.238	0.216
β9	-0.0123	0.0435	-0.282	0.778
β10	-0.0330	0.0440	-0.751	0.453
β11	0.4956	0.2045	2.424	0.016
β12	12.8889	0.0420	306.9	<0.001
β13	0.0982	0.0426	2.304	0.022
β14	0.0057	0.0055	1.039	0.299
β15	-0.0140	0.0055	-2.565	0.011
β16	-0.0045	0.0055	-0.820	0.413
β17	-0.0023	0.0055	-0.420	0.675
β18	0.1802	0.1007	1.790	0.074
β19	11.2787	0.0468	241.1	<0.001
β20	0.0751	0.0468	1.604	0.109
β21	-0.1674	0.0857	-1.953	0.052
β22	11.7763	0.0448	262.7	<0.001
β23	0.0719	0.0450	1.597	0.111

Deviance=422.63 (df=408)

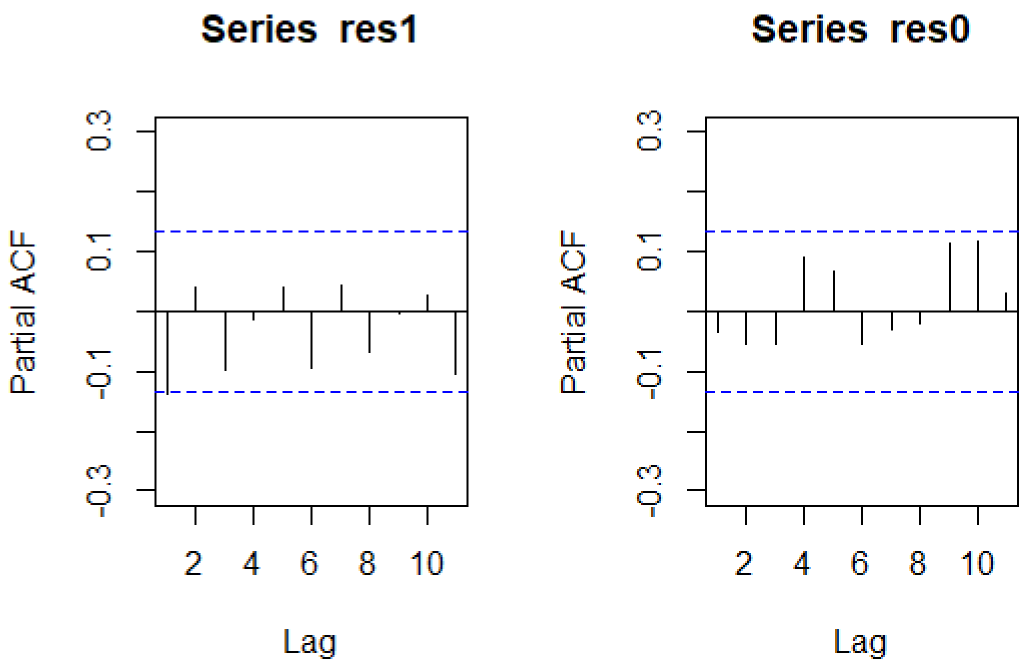

S3_Fig. Plots of the Partial Autocorrelation Function (PACF) for the residuals of perinatal mortality rates from the study region (res1) and control region (res0), for time lags of 1 through 11 months. The horizontal broken lines show the 95% confidence limits

S4_Table. Results of combined regression with Model (2.2)

$$
\begin{aligned}
y(t) \sim (\alpha &+ \exp(\beta1+\beta2\cdot A+\beta3\cdot B+ \beta4\cdot t+\beta5\cdot t\cdot A+\beta6\cdot t\ B+ \\
&cp12\cdot((\beta7\cdot A+\beta8\cdot B+ \beta9\cdot(t-12))\cdot \\
&(1+\beta10\cdot \sin(2\pi\cdot t)+\beta11\cdot \cos(2\pi\cdot t)+\beta12\cdot \sin(4\pi\cdot t)+\beta13\cdot \cos(4\pi\cdot t)))))* \\
&(1+\beta14\cdot \sin(2\pi\cdot t)+\beta15\cdot \cos(2\pi\cdot t)+\beta16\cdot \sin(4\pi\cdot t)+\beta17\cdot \cos(4\pi\cdot t))
\end{aligned}
$$

parameter	estimate	SE	t-value	p-value
α	0.0023	0.0004	6.07	0.000
β1	-5.661	0.086	-65.72	0.000
β2	0.200	0.071	2.82	0.005
β3	0.232	0.046	5.08	0.000
β4	-0.061	0.012	-4.91	0.000
β5	-0.016	0.010	-1.52	0.129
β6	-0.019	0.006	-3.40	0.001
β7	0.276	0.108	2.56	0.011
β8	0.093	0.058	1.60	0.111
β9	0.008	0.013	0.67	0.502
β10	0.453	0.273	1.66	0.097
β11	-0.047	0.218	-0.22	0.829
β12	-0.192	0.224	-0.86	0.391
β13	-0.281	0.237	-1.19	0.236

β14	0.008	0.006	1.41	0.158
β15	-0.015	0.005	-2.79	0.005
β16	-0.005	0.006	-0.86	0.391
β17	-0.001	0.005	-0.26	0.796

Deviance=683.76 (df=630)

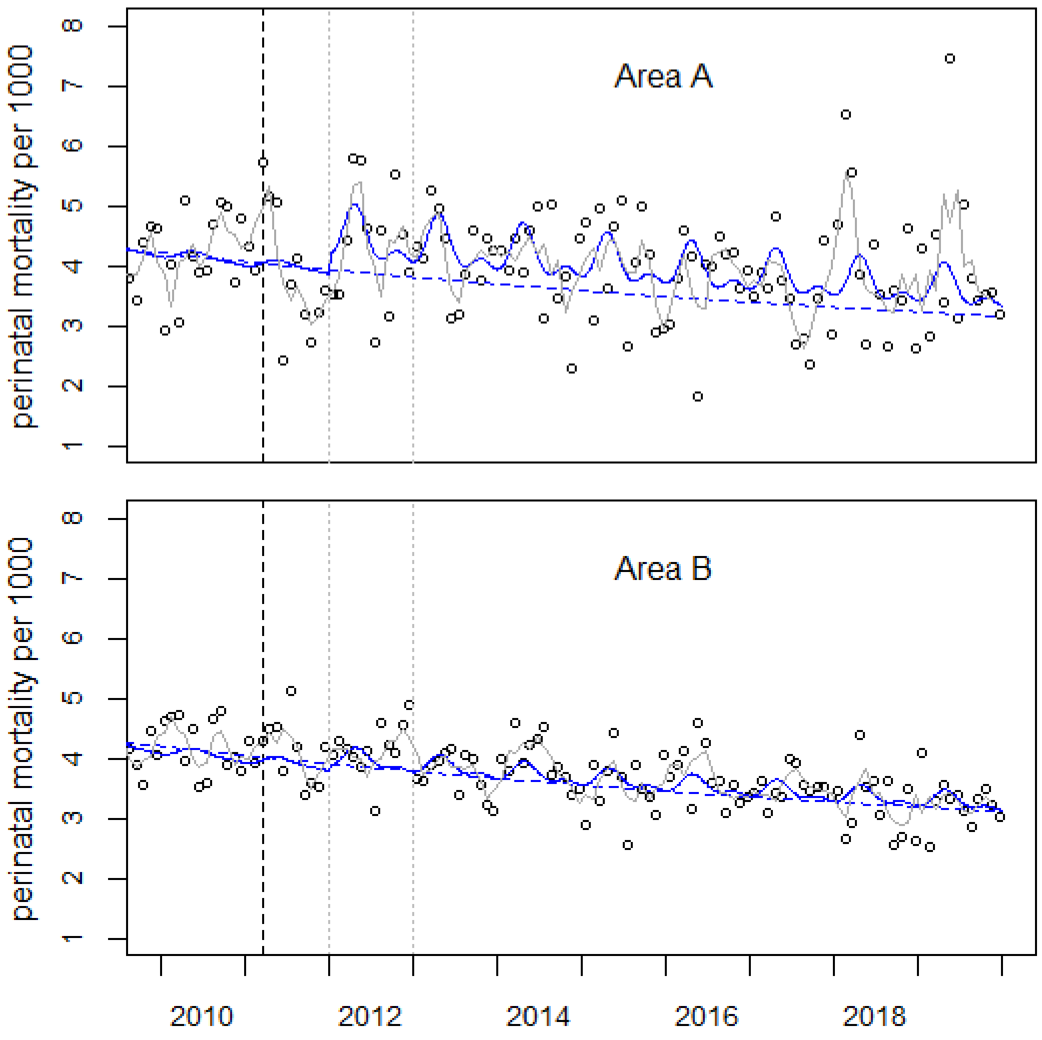

S4_Fig. Perinatal mortality rates in areas A and B and result of a combined regression with Model (2.2) allowing for seasonal effects and periodic peaks in 2012-2019 (solid blue lines). The broken blue lines show the predicted undisturbed trends.

S5_Table. Results of combined regression with model (2.3)

```
y(t) ~ (α +exp(β1+β2·A+β3·B+ β4·t+β5·t·A+β6·t B+cp12·((β7·A+β8·B+β9·(t-12))
       (1+β10·sin(2π·t)+β11·cos(2π·t)+β12·sin(4π·t)+β13·cos(4π·t))+
       β14·A cp18+(β15·A+β16·B)/exp((t-β17)^2/2/β18^2))))*
       (1+β19·sin(2π·t)+β20·cos(2π·t)+β21·sin(4π·t)+β22·cos(4π·t)+
       (β23·A+β24·B)/exp((t-β25)^2/2/β18^2)+(β26·A+β27·B)/exp((t-β28)^2/2/β18^2))
```

parameter	estimate	SE	t-value	p-value
α	0.0023	0.0003	6.88	0.0000
β1	-5.674	0.078	-72.90	0.0000
β2	0.236	0.074	3.18	0.0016
β3	0.229	0.045	5.04	0.0000
β4	-0.063	0.012	-5.27	0.0000
β5	-0.022	0.011	-1.92	0.0550
β6	-0.019	0.006	-3.22	0.0014
β7	0.275	0.115	2.40	0.0169
β8	0.095	0.058	1.66	0.0983
β9	0.002	0.013	0.14	0.8877
β10	0.592	0.373	1.59	0.1132
β11	-0.147	0.274	-0.54	0.5927
β12	-0.114	0.251	-0.46	0.6496
β13	-0.392	0.306	-1.28	0.2004
β14	0.374	0.165	2.27	0.0237
β15	0.292	0.388	0.75	0.4522
β16	0.519	0.209	2.48	0.0133
β17	12.917	0.032	398.37	0.0000
β18	0.071	0.022	3.18	0.0016
β19	0.006	0.006	1.10	0.2723
β20	-0.016	0.005	-2.88	0.0041
β21	-0.005	0.005	-0.89	0.3762
β22	-0.001	0.005	-0.26	0.7956
β23	0.437	0.220	1.99	0.0474
β24	0.116	0.097	1.19	0.2337
β25	11.264	0.038	295.57	0.0000
β26	-0.310	0.154	-2.02	0.0440
β27	-0.130	0.086	-1.51	0.1325
β28	11.782	0.041	288.99	0.0000

Deviance=652.20 (df=619)

S6_Table. Results of combined regression with Model (2.4)

```
y(t) ~ (α +exp(β1+β2·study+β4·t+β5·t·study+cp12·((β7·A+β8·B)
       (1+β10·sin(2π·t)+β11·cos(2π·t)+β12·sin(4π·t)+β13·cos(4π·t))+
        β14·A cp18+(β15·A+β16·B)/exp((t-β17)^2/2/β18^2))))*
       (1+β19·sin(2π·t)+β20·cos(2π·t)+β21·sin(4π·t)+β22·cos(4π·t)+
       (β23·A+β24·B)/exp((t-β25)^2/2/β18^2)+(β26·A+β27·B)/exp((t-β28)^2/2/β18^2))
```

parameter	estimate	SE	t-value	p-value
α	0.0024	0.0003	8.28	0.0000
β1	-5.683	0.066	-85.55	0.0000
β2	0.229	0.043	5.37	0.0000
β4	-0.065	0.011	-6.06	0.0000
β5	-0.019	0.006	-3.42	0.0007
β7	0.251	0.085	2.94	0.0034
β8	0.100	0.053	1.91	0.0571
β10	0.660	0.385	1.71	0.0872
β11	-0.185	0.306	-0.60	0.5458
β12	-0.117	0.277	-0.42	0.6742
β13	-0.442	0.325	-1.36	0.1753
β14	0.375	0.140	2.67	0.0077
β15	0.304	0.393	0.78	0.4388
β16	0.541	0.208	2.60	0.0096
β17	12.917	0.032	406.1	0.0000
β18	0.071	0.022	3.21	0.0014
β19	0.006	0.005	1.09	0.2767
β20	-0.015	0.005	-2.86	0.0043
β21	-0.005	0.005	-0.89	0.3738
β22	-0.001	0.005	-0.25	0.8038
β23	0.417	0.210	1.99	0.0472
β24	0.117	0.097	1.22	0.2246
β25	11.265	0.039	288.6	0.0000
β26	-0.321	0.149	-2.15	0.0319
β27	-0.128	0.085	-1.51	0.1327
β28	11.782	0.040	296.4	0.0000

Deviance=651.95 (df=622)

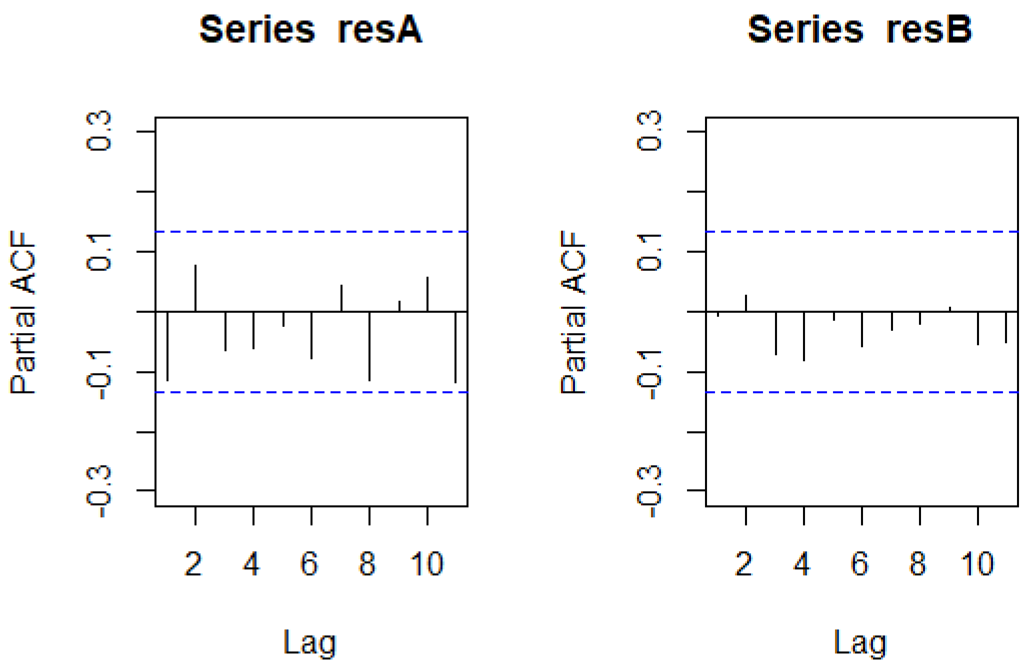

S5_Fig. Plots of the Partial Autocorrelation Function (PACF) for the residuals of perinatal mortality rates from area A (resA) and area B (resB), for time lags of 1 through 11 months. The horizontal broken lines show the 95% confidence limits.

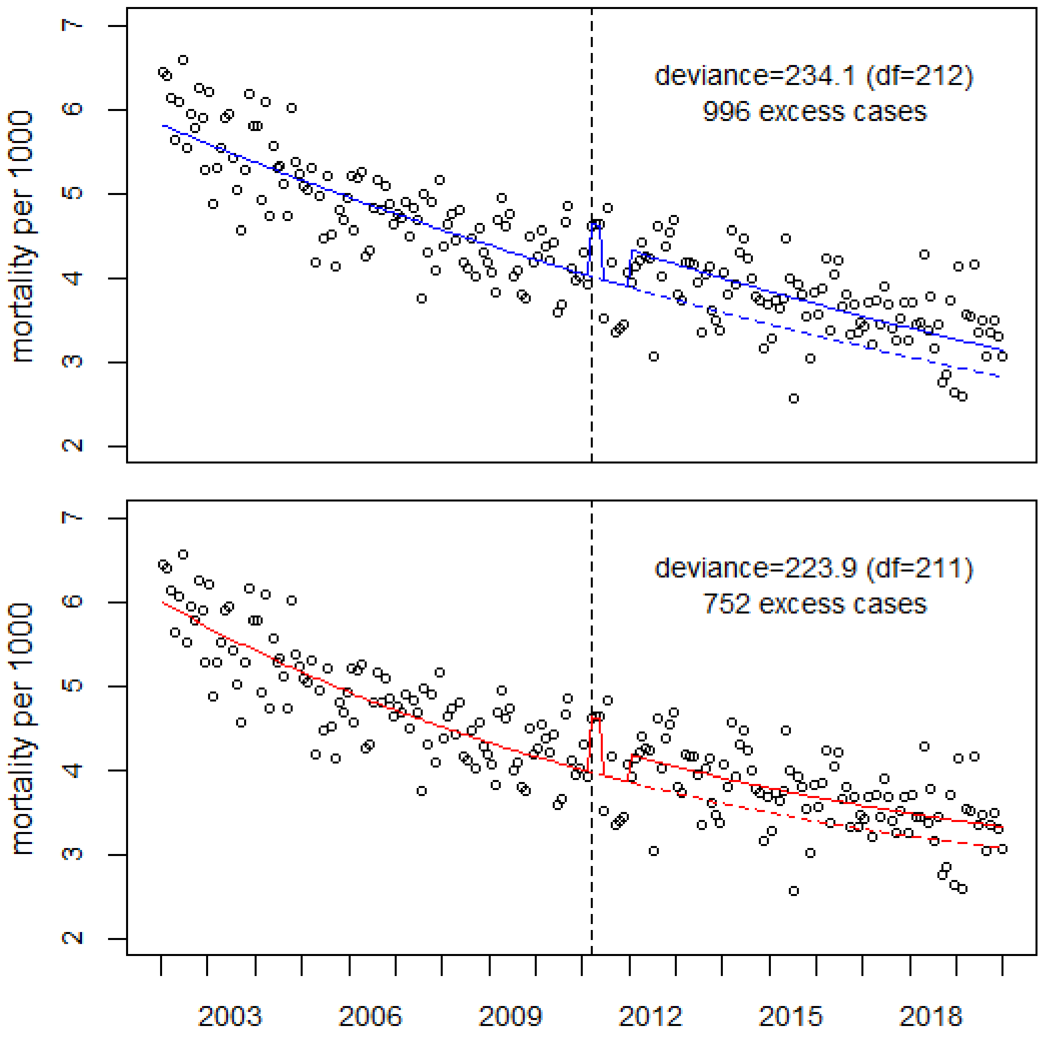

S6_Fig. Perinatal mortality in the study region (12 prefectures) and results of logistic regressions with a linear (upper panel) and a linear-quadratic (lower panel) time trend. The regression allows for a level change in January 2012 and a peak in March-May 2011.

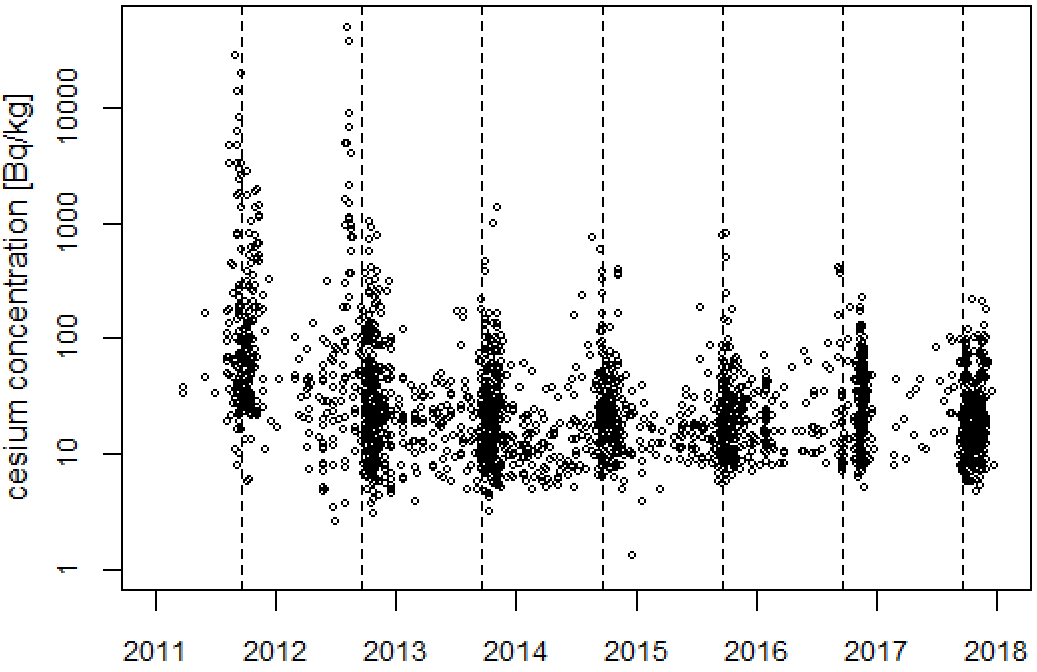

S7_Fig. Cesium concentration (Cs-134 plus Cs-137) in mushrooms from Fukushima Prefecture plus four neighboring prefectures, 2011-2017, semi-logarithmic plot. The vertical broken lines denote mid-September.

Data Source:

Levels of Radioactive Contaminants in Foods Tested in Respective Prefectures. https://www.mhlw.go.jp/english/topics/2011eq/index_food_radioactive.html).

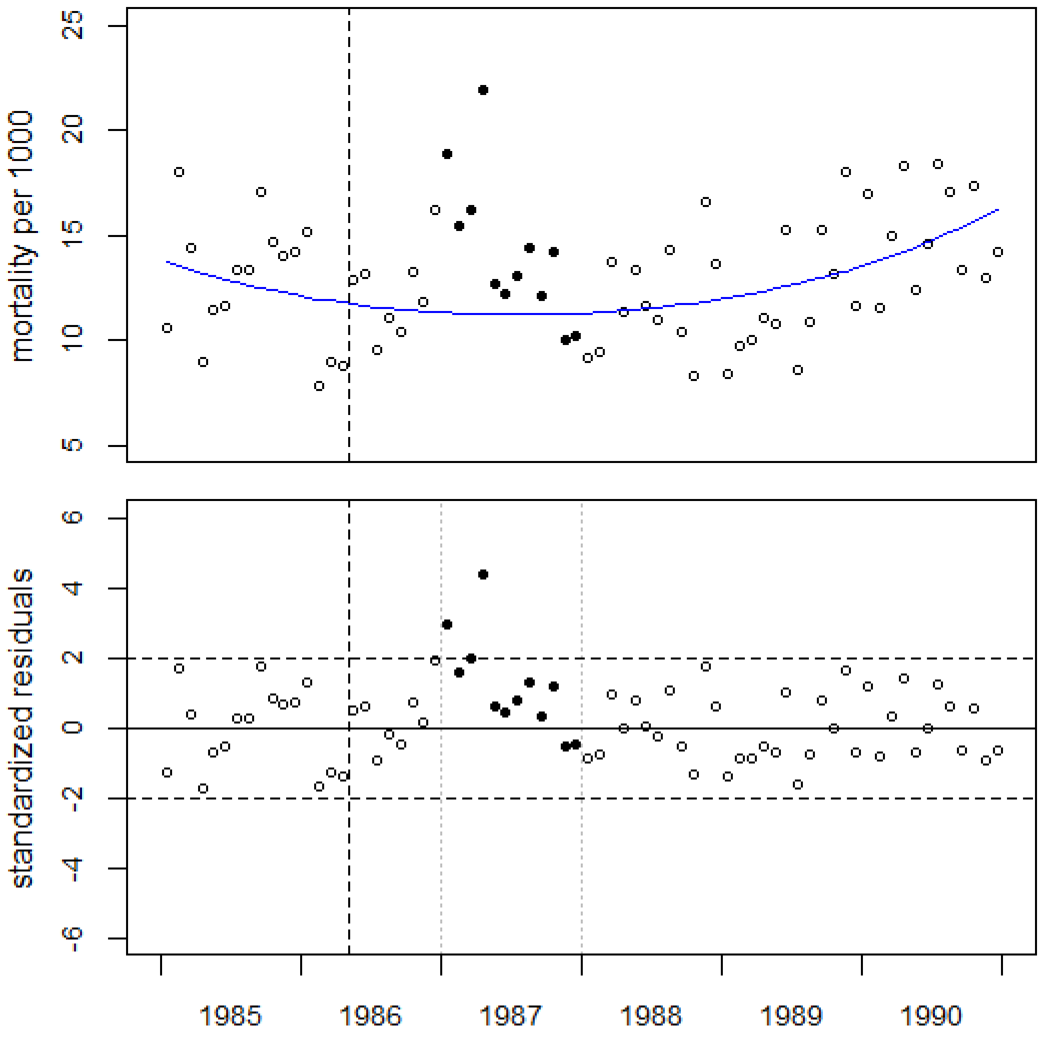

S8_Fig. Perinatal mortality in Oblast Zhytomyr (Ukraine) and result of a linear-quadratic logistic regression without the data from 1987. The lower panel shows the residuals in units of standard deviations (standardized residuals). The vertical broken line indicates the date of the Chernobyl accident. The black circles mark the data from 1987.

S7_Table. Data of perinatal mortality in oblast Zhytomyr, Ukraine, 1985-1990

year	LB	SB	NEO7	year	LB	SB	NEO7
1985.042	2075	16	6	1988.042	1742	9	7
1985.125	1877	25	9	1988.125	1796	11	6
1985.208	2066	20	10	1988.208	1879	14	12
1985.292	2113	16	3	1988.292	1839	19	2
1985.375	2257	14	12	1988.375	2086	19	9
1985.458	2131	17	8	1988.458	1965	15	8
1985.542	2232	21	9	1988.542	2080	13	10
1985.625	2158	17	12	1988.625	1880	17	10
1985.708	1861	19	13	1988.708	1818	9	10
1985.792	1833	17	10	1988.792	1680	11	3
1985.875	1699	17	7	1988.875	1619	18	9
1985.958	1607	15	8	1988.958	1608	15	7
1986.042	2095	21	11	1989.042	1769	11	4
1986.125	1904	11	4	1989.125	1633	13	3
1986.208	2219	12	8	1989.208	1890	11	8
1986.292	2387	13	8	1989.292	1794	10	10
1986.375	2322	24	6	1989.375	1936	13	8
1986.458	2042	18	9	1989.458	1828	20	8
1986.542	2191	17	4	1989.542	1850	11	5
1986.625	1979	15	7	1989.625	1828	8	12
1986.708	1908	12	8	1989.708	1622	12	13
1986.792	1873	18	7	1989.792	1658	15	7
1986.875	1856	13	9	1989.875	1595	16	13
1986.958	1713	16	12	1989.958	1711	15	5
1987.042	1738	21	12	1990.042	1752	20	10
1987.125	1674	15	11	1990.125	1643	13	6
1987.208	1837	17	13	1990.208	1667	18	7
1987.292	1851	25	16	1990.292	1681	20	11
1987.375	1956	11	14	1990.375	1763	15	7
1987.458	1948	13	11	1990.458	1702	13	12
1987.542	1975	16	10	1990.542	1780	21	12
1987.625	1856	15	12	1990.625	1690	16	13
1987.708	1722	13	8	1990.708	1486	14	6
1987.792	1753	16	9	1990.792	1484	16	10
1987.875	1694	10	7	1990.875	1451	8	11
1987.958	1759	12	6	1990.958	1535	11	11

LB: Live births, SB: stillbirths, NEO7: Early neonatal deaths (first seven days)

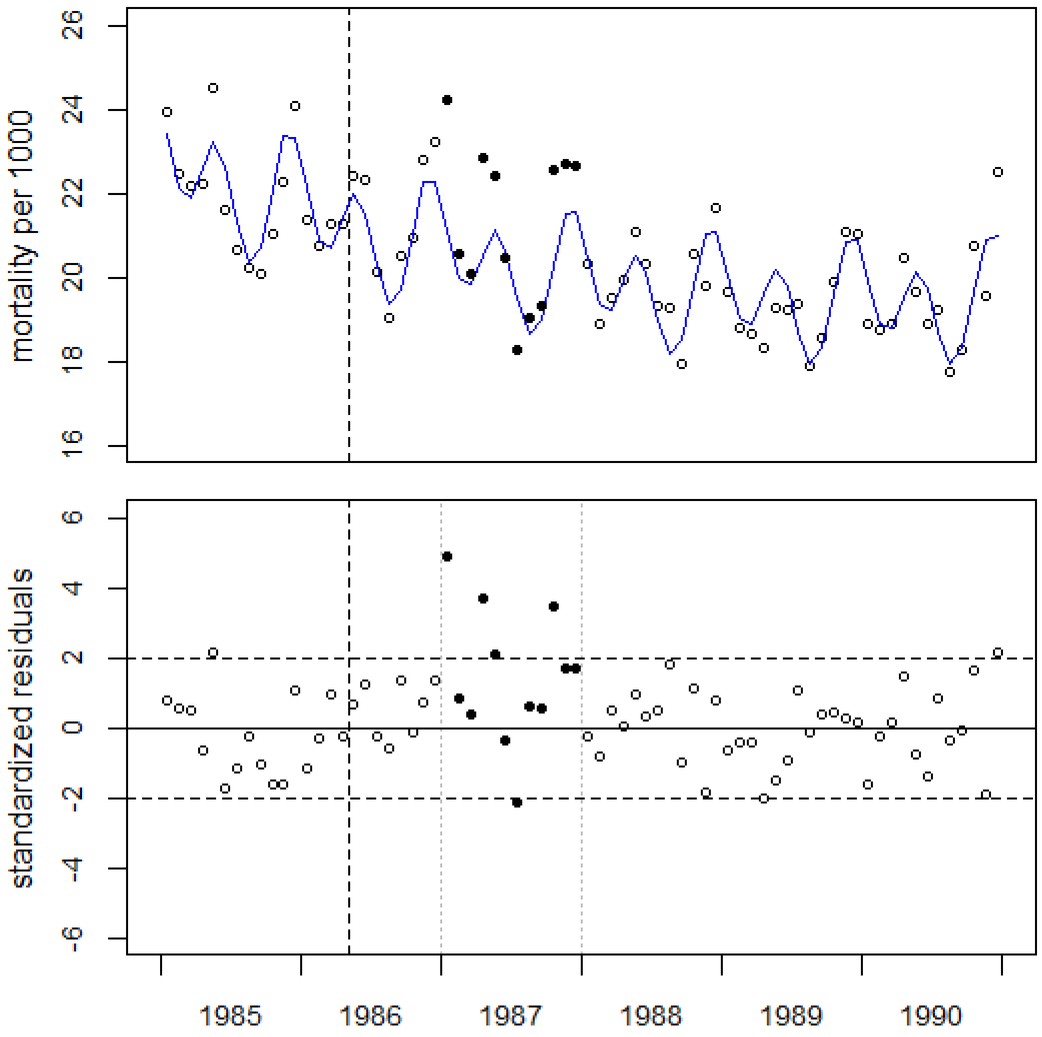

S9_Fig. Infant mortality Poland and result of a linear-quadratic logistic regression without the data from 1987. The regression model allowed for seasonal variations. The lower panel shows the standardized residuals.

S8_Table. Data of infant mortality in Poland, 1985-1990

year	LB	ID	year	LB	ID
1985.042	59370	1420	1988.042	51349	1043
1985.125	54188	1218	1988.125	48625	919
1985.209	62924	1396	1988.209	54047	1056
1985.292	59505	1323	1988.292	50040	999
1985.375	60446	1482	1988.375	53158	1122
1985.459	56790	1227	1988.459	49685	1010
1985.542	60658	1253	1988.542	51805	1000
1985.625	57238	1159	1988.625	49610	957
1985.709	55542	1115	1988.709	47838	858
1985.792	52675	1108	1988.792	45725	940
1985.875	49424	1102	1988.875	43359	858
1985.959	51331	1235	1988.959	44697	967
1986.042	55359	1184	1989.042	49439	971
1986.125	51425	1067	1989.125	45903	862
1986.209	57903	1232	1989.209	49718	928
1986.292	57327	1219	1989.292	47605	873
1986.375	56083	1257	1989.375	49308	950
1986.459	53542	1195	1989.459	48440	932
1986.542	56988	1148	1989.542	50291	975
1986.625	53113	1012	1989.625	47886	856
1986.709	51856	1065	1989.709	46541	863
1986.792	49740	1042	1989.792	45397	903
1986.875	45532	1039	1989.875	41724	879
1986.959	48345	1122	1989.959	42179	888
1987.042	51413	1246	1990.042	48318	912
1987.125	47525	977	1990.125	43985	825
1987.209	52644	1058	1990.209	48872	923
1987.292	51249	1172	1990.292	45391	929
1987.375	52753	1183	1990.375	47481	933
1987.459	53934	1103	1990.459	45508	859
1987.542	56070	1024	1990.542	48913	940
1987.625	52210	994	1990.625	48126	854
1987.709	48290	933	1990.709	45319	828
1987.792	46407	1048	1990.792	42245	877
1987.875	45582	1034	1990.875	41101	805

LB: Live births, ID: infant deaths

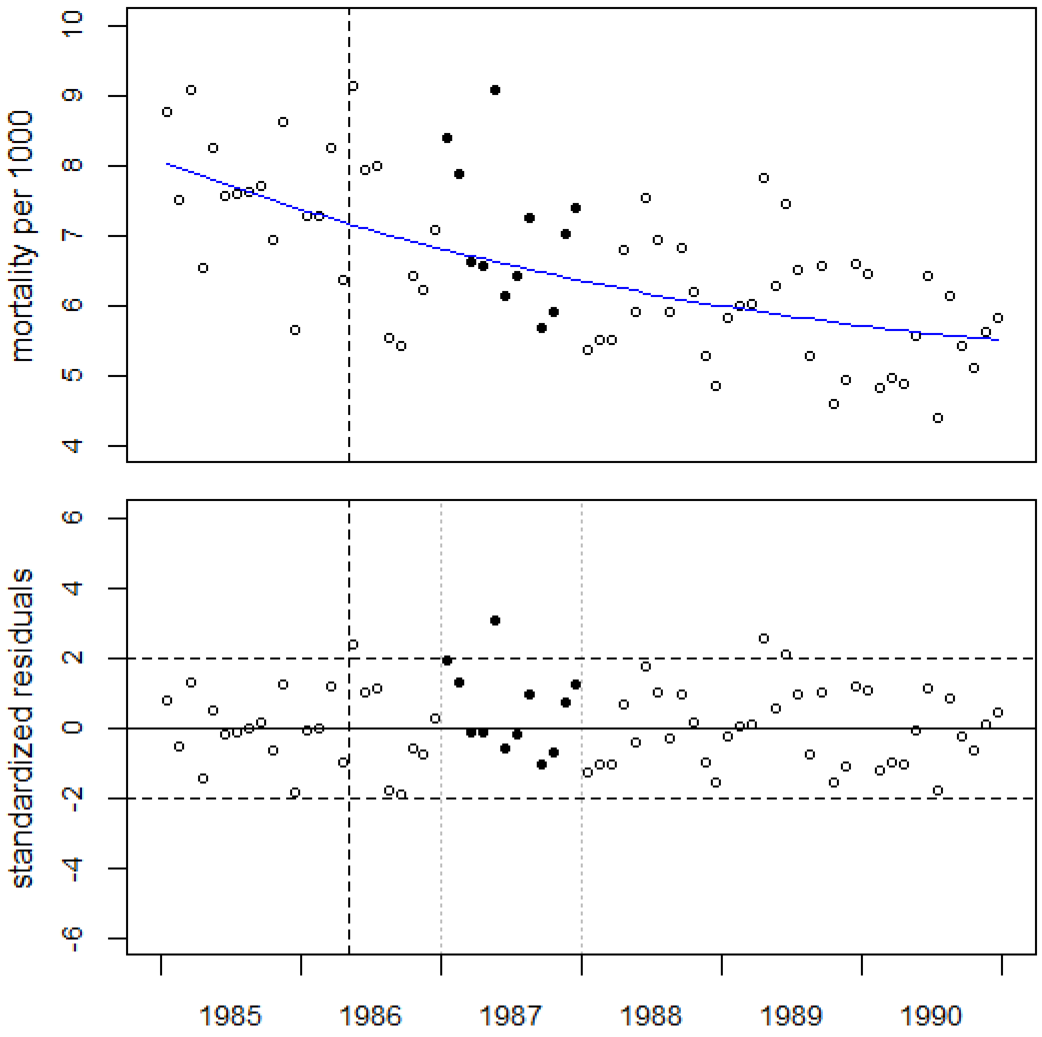

S10_Fig. Perinatal mortality in Bavaria, 1985-1990, and result of a linear-quadratic logistic regression without the data from 1987. The lower panel shows the standardized residuals.

S9_Table. Data of perinatal mortality in Bavaria, 1985-1990

year	LB	SB	NEO7	year	LB	SB	NEO7
1985.042	9667	50	35	1988.042	10598	39	18
1985.125	9435	38	33	1988.125	10312	31	26
1985.208	9779	52	37	1988.208	11197	31	31
1985.292	9293	31	30	1988.292	9844	41	26
1985.375	9781	39	42	1988.375	10968	45	20
1985.458	8963	33	35	1988.458	10179	42	35
1985.542	9860	42	33	1988.542	10799	50	25
1985.625	9277	44	27	1988.625	11144	33	33
1985.708	9306	33	39	1988.708	10950	41	34
1985.792	9465	34	32	1988.792	10305	38	26
1985.875	8436	49	24	1988.875	9817	28	24
1985.958	8103	23	23	1988.958	10296	27	23
1986.042	9718	32	39	1989.042	10460	27	34
1986.125	9169	36	31	1989.125	10131	27	34
1986.208	9890	45	37	1989.208	10927	34	32
1986.292	9856	33	30	1989.292	10174	41	39
1986.375	10020	49	43	1989.375	10790	38	30
1986.458	9769	34	44	1989.458	10449	43	35
1986.542	10358	43	40	1989.542	11645	40	36
1986.625	10268	33	24	1989.625	11114	28	31
1986.708	10642	30	28	1989.708	10799	37	34
1986.792	9919	31	33	1989.792	10214	26	21
1986.875	9122	26	31	1989.875	9925	30	19
1986.958	9708	40	29	1989.958	10401	28	41
1987.042	9964	43	41	1990.042	11256	36	37
1987.125	8965	41	30	1990.125	10734	28	24
1987.208	9944	36	30	1990.208	11439	33	24
1987.292	9739	40	24	1990.292	10632	30	22
1987.375	10321	50	44	1990.375	11445	38	26
1987.458	10089	32	30	1990.458	10550	40	28
1987.542	11038	39	32	1990.542	12222	27	27
1987.625	10577	41	36	1990.625	12366	45	31
1987.708	10538	24	36	1990.708	12332	28	39
1987.792	9634	35	22	1990.792	11522	36	23
1987.875	9376	44	22	1990.875	10832	29	32
1987.958	9438	36	34	1990.958	10792	37	26

LB: Live births, SB: stillbirths, NEO7: Early neonatal deaths (first seven days)

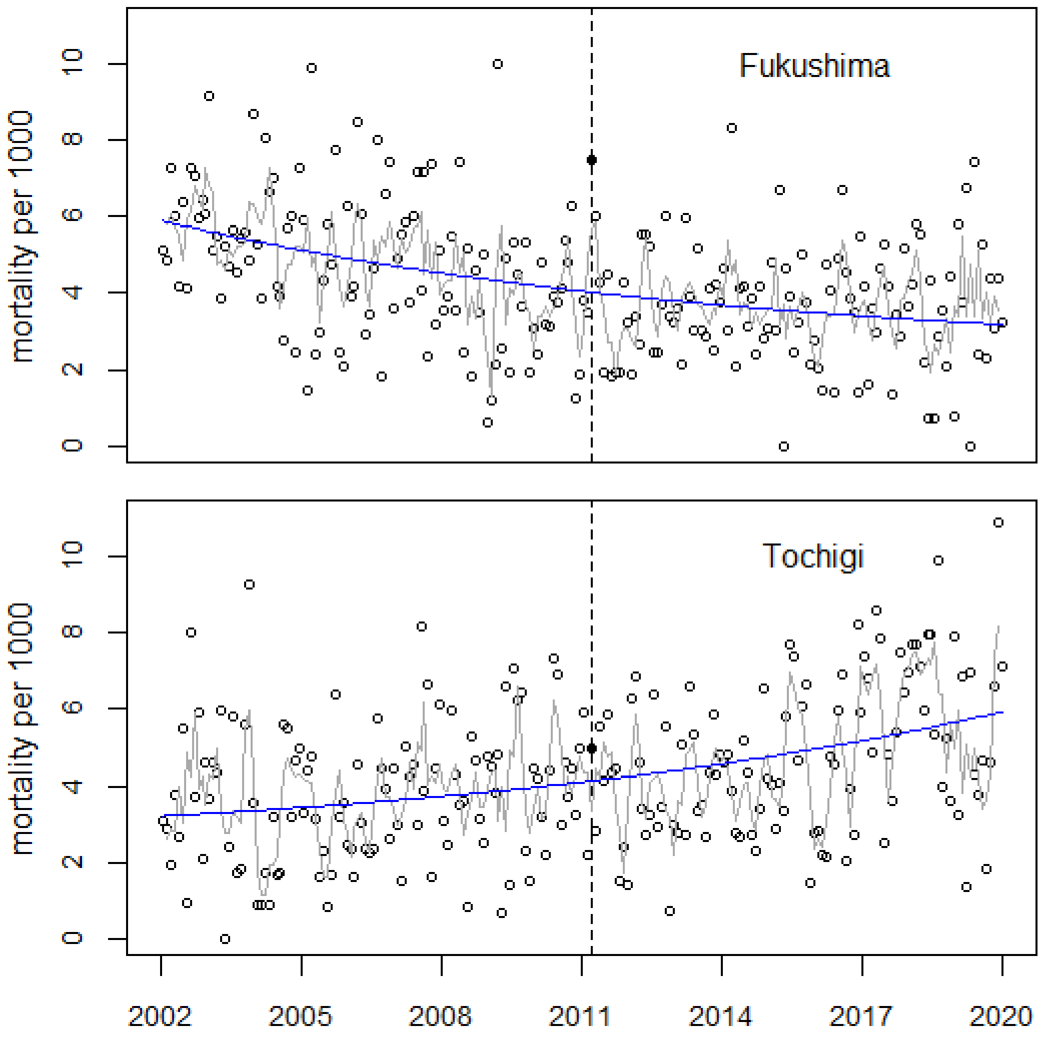

S11_Fig. Monthly perinatal mortality rates in Fukushima prefecture and the inland prefecture Tochigi, and 3-month moving average (grey line) and regression lines. In March 2011 (vertical broken line), there is a significant increase (+84%, p=0.023) in Fukushima Prefecture, but no notable increase in the inland prefecture Tochigi (+20%, p=0.61).

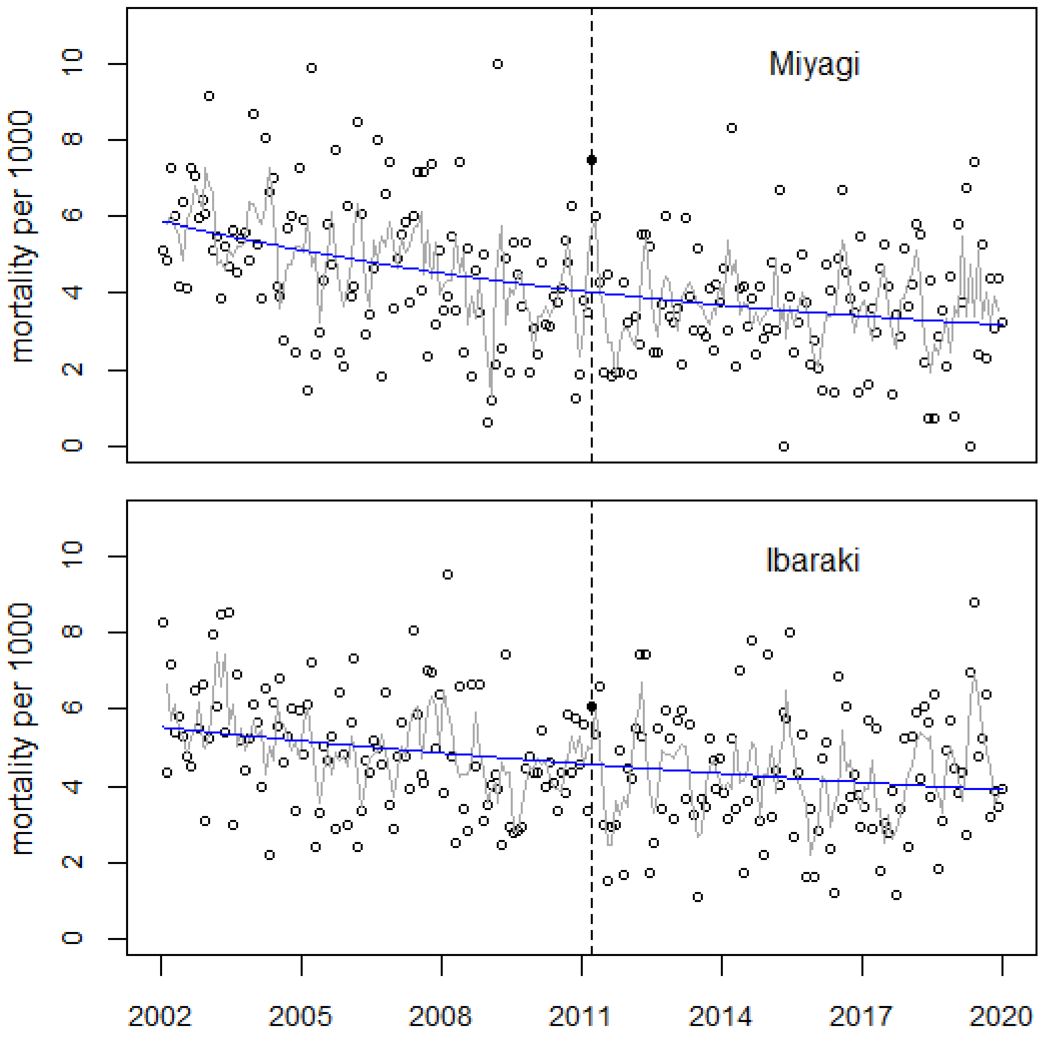

S12_Fig. Monthly perinatal mortality rates in the two coastal prefectures Miyagi and Ibaraki and 3-month moving average (black line), and regression lines. In March 2011 (vertical broken line), there is a significant increase (+85%, p=0.024) in Miyagi and a non-significant increase in Ibaraki (+30%, p=0.30).

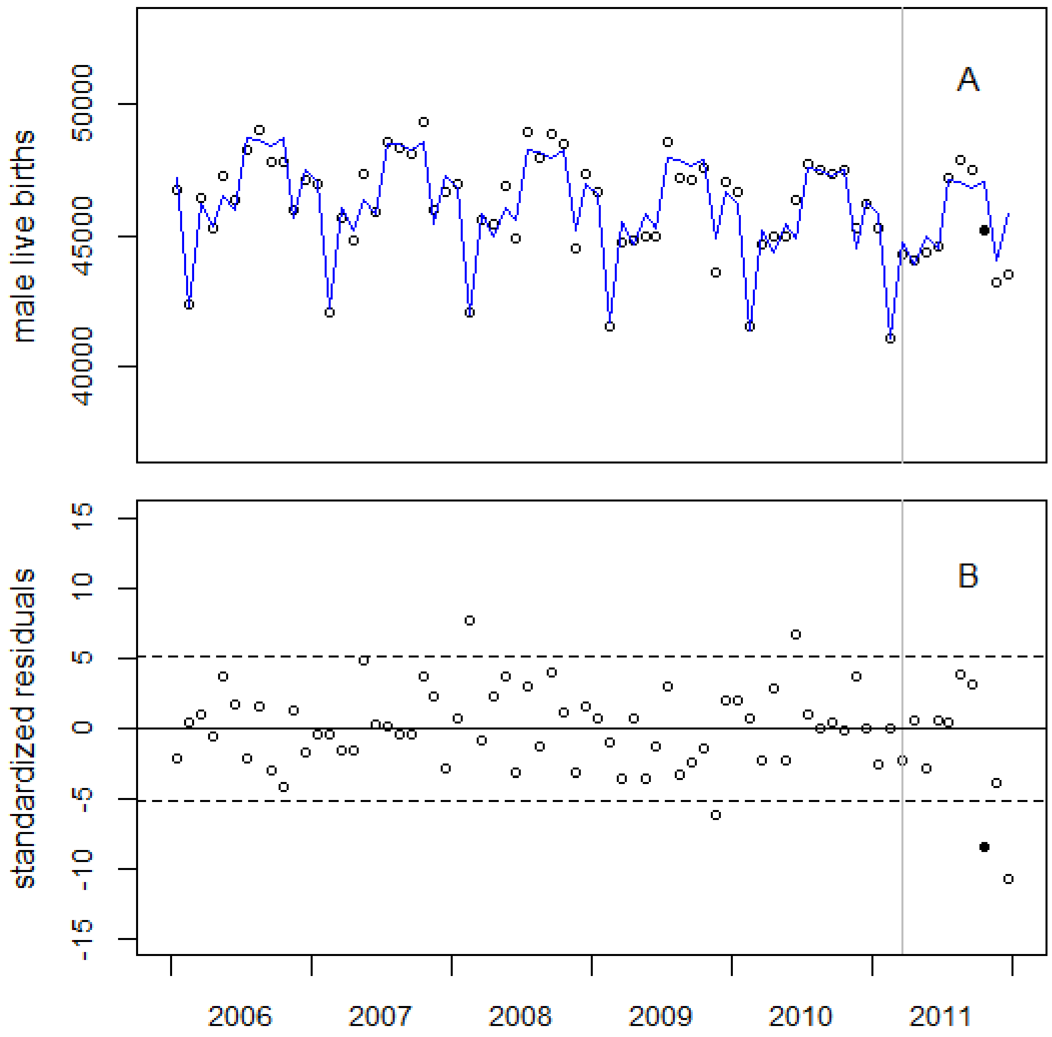

S13_Fig. Panel A: Trend of **male** live births in Japan, 2006-2011 and regression line.
Panel B: Residuals in units of standard deviations (standardized residuals).

A statistically significant drop is observed in October 2011 [-3.9% (-6.3%, -1.4%), p=0.003].

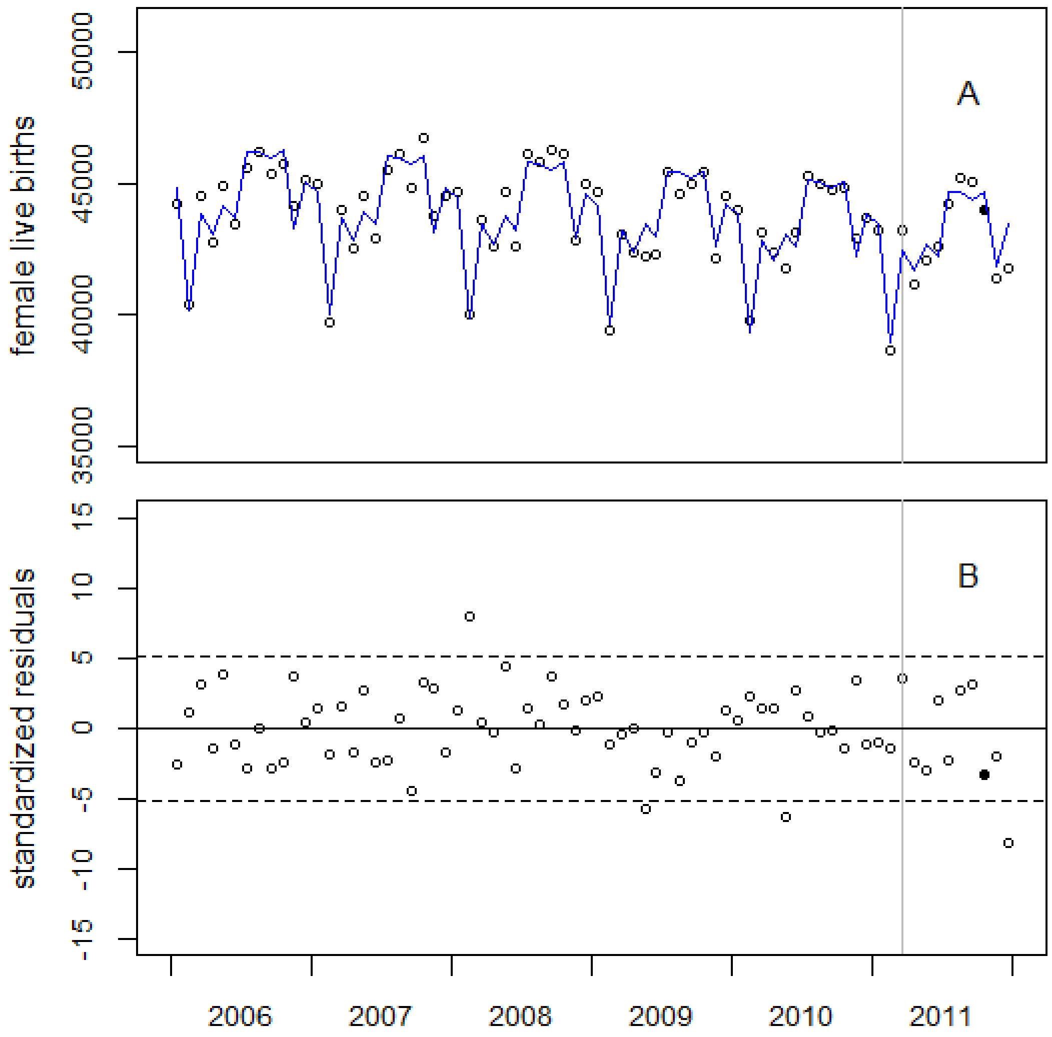

S14_Fig. Panel A: Trend of **female** live births in Japan, 2006-2011 and regression line. Panel B: Residuals in units of standard deviations (standardized residuals).

The drop in October 2011 is not statistically significant [-1.5%, p=0.25].

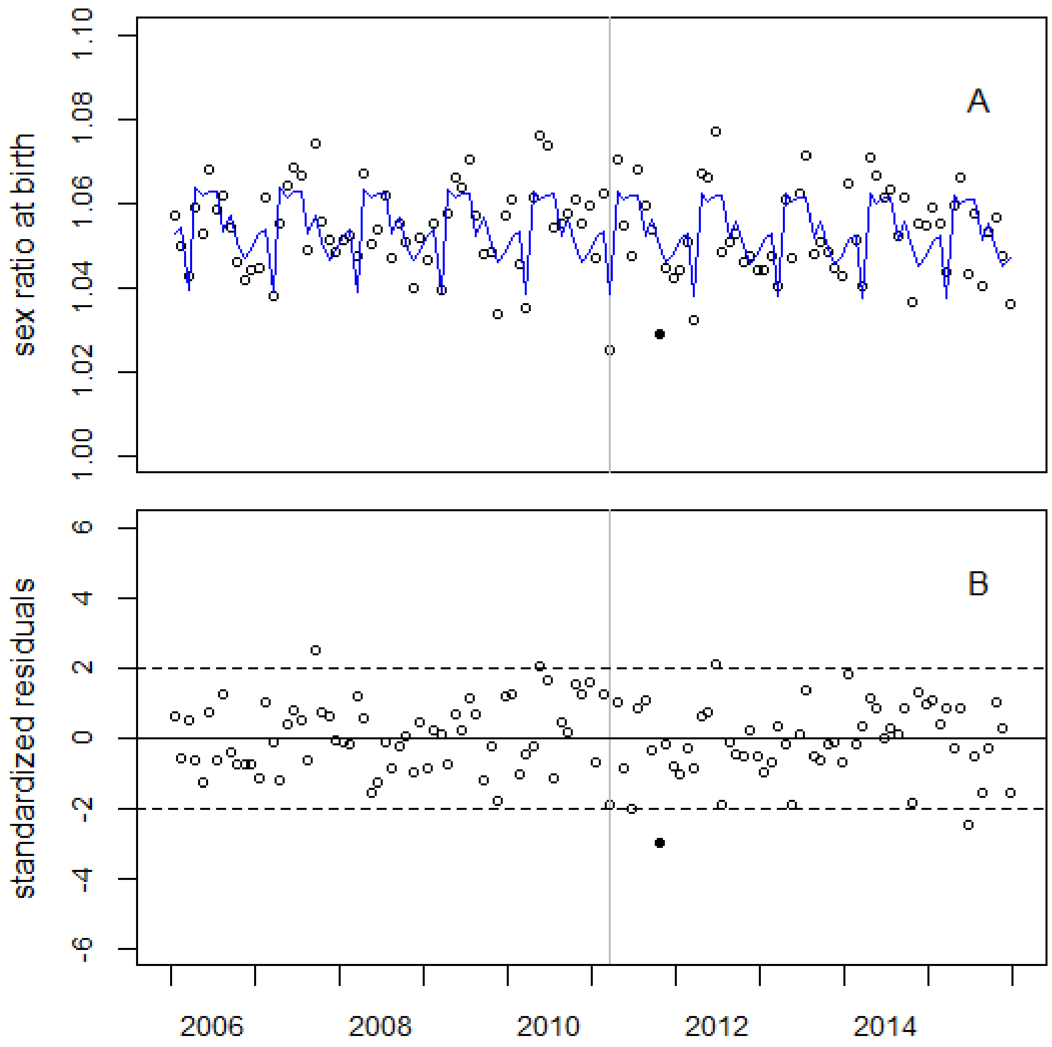

S15_Fig. Panel A: Trend of sex ratios at birth (ratio of male to female births) in Japan, 2006-2015, and regression line. Panel B: Residuals in units of standard deviations (standardized residuals)

In October 2011, the sex ratio is 2% lower than expected [-2.0% (-3.4%, -0.6%), p=0.008].

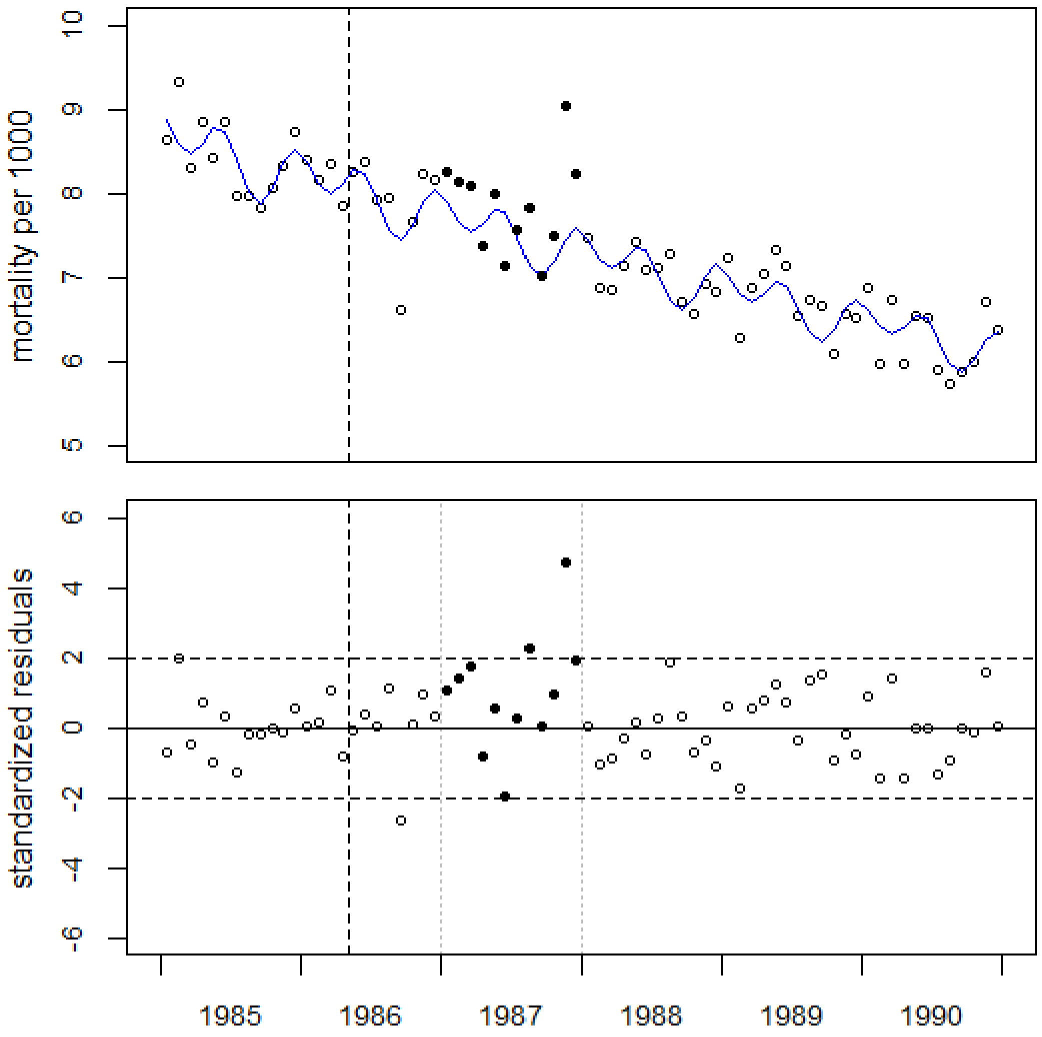

S16_Fig. Perinatal mortality in Germany and result of a linear-quadratic logistic regression without the data from 1987 and allowing for seasonal variations. The lower panel shows the standardized residuals.

S10_Table. Data of perinatal mortality in Germany, 1985-1990

year	LB	SB	NEO7	year	LB	SB	NEO7
1985.042	68732	302	294	1988.042	75114	311	253
1985.125	63118	296	295	1988.125	71349	265	228
1985.208	70931	308	284	1988.208	78060	294	243
1985.292	68183	318	288	1988.292	70574	266	239
1985.375	70134	303	291	1988.375	76674	306	265
1985.458	65523	297	286	1988.458	72840	285	233
1985.542	72341	310	269	1988.542	77328	293	260
1985.625	69628	314	243	1988.625	78452	319	254
1985.708	70834	287	269	1988.708	78656	309	221
1985.792	68346	289	264	1988.792	72651	263	215
1985.875	63045	282	246	1988.875	69743	278	207
1985.958	62988	295	258	1988.958	71552	285	205
1986.042	70286	289	303	1989.042	72834	275	253
1986.125	64858	295	237	1989.125	69245	211	225
1986.208	70407	305	286	1989.208	75143	270	249
1986.292	71422	316	247	1989.292	71312	275	230
1986.375	71707	307	288	1989.375	74690	307	242
1986.458	70263	273	317	1989.458	72707	283	238
1986.542	73911	318	271	1989.542	78785	281	236
1986.625	73132	299	284	1989.625	77921	300	227
1986.708	75288	269	231	1989.708	74803	268	233
1986.792	71459	287	262	1989.792	71869	250	190
1986.875	65403	272	269	1989.875	69013	265	190
1986.958	70096	317	258	1989.958	72137	262	210
1987.042	71684	312	282	1990.042	75800	291	233
1987.125	65940	259	279	1990.125	70491	242	181
1987.208	73254	316	280	1990.208	76630	281	237
1987.292	70554	271	252	1990.292	72614	244	192
1987.375	74353	299	297	1990.375	76566	268	235
1987.458	75144	295	244	1990.458	73072	275	203
1987.542	78254	314	280	1990.542	81098	278	202
1987.625	75752	333	263	1990.625	81563	275	194
1987.708	75833	293	242	1990.708	80349	255	219
1987.792	70924	285	248	1990.792	76756	263	198
1987.875	67007	319	289	1990.875	70483	269	206
1987.958	69270	306	266	1990.958	70253	261	188

LB: Live births, SB: stillbirths, NEO7: Early neonatal deaths (first seven days)
